# Transcriptomic profiling of early synucleinopathy in rats induced with preformed fibrils

**DOI:** 10.1038/s41531-023-00620-y

**Published:** 2024-01-03

**Authors:** Joseph R. Patterson, Joseph Kochmanski, Anna C. Stoll, Michael Kubik, Christopher J. Kemp, Megan F. Duffy, Kajene Thompson, Jacob W. Howe, Allyson Cole-Strauss, Nathan C. Kuhn, Kathryn M. Miller, Seth Nelson, Christopher U. Onyekpe, John S. Beck, Scott E. Counts, Alison I. Bernstein, Kathy Steece-Collier, Kelvin C. Luk, Caryl E. Sortwell

**Affiliations:** 1 Department of Translational Science and Molecular Medicine, Michigan State University, Grand Rapids, MI USA; 2 Neuroscience Program, Michigan State University, East Lansing, MI USA; 3 Department of Pharmacology and Toxicology, Michigan State University, East Lansing, MI USA; 4 Department of Pharmacology and Toxicology, Rutgers University, Piscataway, NJ USA; 5 Environmental and Occupational Health Science Institute, Rutgers University, Piscataway, NJ USA; 6 Center for Neurodegenerative Disease Research, Department of Pathology and Laboratory Medicine, University of Pennsylvania Perelman School of Medicine, Philadelphia, PA USA

**Keywords:** Parkinson's disease, Synaptic transmission

## Abstract

Examination of early phases of synucleinopathy when inclusions are present, but long before neurodegeneration occurs, is critical to both understanding disease progression and the development of disease modifying therapies. The rat alpha-synuclein (α-syn) preformed fibril (PFF) model induces synchronized synucleinopathy that recapitulates the pathological features of Parkinson’s disease (PD) and can be used to study synucleinopathy progression. In this model, phosphorylated α-syn (pSyn) inclusion-containing neurons and reactive microglia (major histocompatibility complex-II immunoreactive) peak in the substantia nigra pars compacta (SNpc) months before appreciable neurodegeneration. However, it remains unclear which specific genes are driving these phenotypic changes. To identify transcriptional changes associated with early synucleinopathy, we used laser capture microdissection of the SNpc paired with RNA sequencing (RNASeq). Precision collection of the SNpc allowed for the assessment of differential transcript expression in the nigral dopamine neurons and proximal glia. Transcripts upregulated in early synucleinopathy were mainly associated with an immune response, whereas transcripts downregulated were associated with neurotransmission and the dopamine pathway. A subset of 29 transcripts associated with neurotransmission/vesicular release and the dopamine pathway were verified in a separate cohort of males and females to confirm reproducibility. Within this subset, fluorescent in situ hybridization (*FISH*) was used to localize decreases in the *Syt1* and *Slc6a3* transcripts to pSyn inclusion-containing neurons. Identification of transcriptional changes in early synucleinopathy provides insight into the molecular mechanisms driving neurodegeneration.

## Introduction

Accumulation of Lewy pathology, subsequent progressive neurodegeneration of the nigrostriatal pathway, and motor symptoms are key hallmarks of Parkinson’s disease (PD). Significant neurodegeneration occurs prior to motor impairment, with an estimated 50-80% loss of nigrostriatal dopamine terminals in the putamen^1–4^ and 30–50% loss of the corresponding neuron soma in the substantia nigra (SN)^3,5–8^ by the onset of motor symptoms. Individuals with PD can live for decades as neurodegeneration progresses. Only upon postmortem examination is an estimated loss of 60-80% of dopamine neurons (DaNs) in the SN directly observed and diagnosis confirmed^5,9,10^. Currently, symptomatic treatments for PD are available but no disease-modifying strategies exist. In order to develop disease-modifying strategies, a better understanding of the early pathogenic mechanisms associated with the development of Lewy pathology prior to degeneration is required.

Previous studies examining gene expression in postmortem PD nigra implicate alterations in pathways associated with mitochondrial function, the ubiquitin-proteosome system and proteolysis, inflammation, signal transduction, ion transport, chaperone processes, cytoskeleton and axonal guidance, and dopaminergic neurotransmission^11–23^. *SNCA* transcript levels are also altered in PD SN compared to controls. However, results pertaining to the directionality of transcriptional change are conflicting, with some groups showing an increase^18^ and others showing a decrease^23^. Interpretation of these results is additionally confounded by limitations associated with the use of postmortem human samples. PD subjects have different underlying genetics, age, diets, health issues, and environmental exposures, all of which can confound disease-related associations with transcription. Furthermore, postmortem PD tissue can only provide gene expression levels at a single time point in a decades-long, chronic disease, and brain samples are most frequently collected from symptomatic individuals at later disease stages, when they exhibit mixed Lewy, neuroinflammatory, and degeneration co-pathologies. Depending on the approach, sampled nigral DaNs may or may not have Lewy pathology, and analysis of late-stage nigral tissue may skew toward surviving nigral neurons lacking Lewy pathology altogether. The ability to delineate early Lewy body-associated gene expression changes from overt neurodegeneration is imperative to identifying potential disease-modifying targets.

**Fig. 1:**
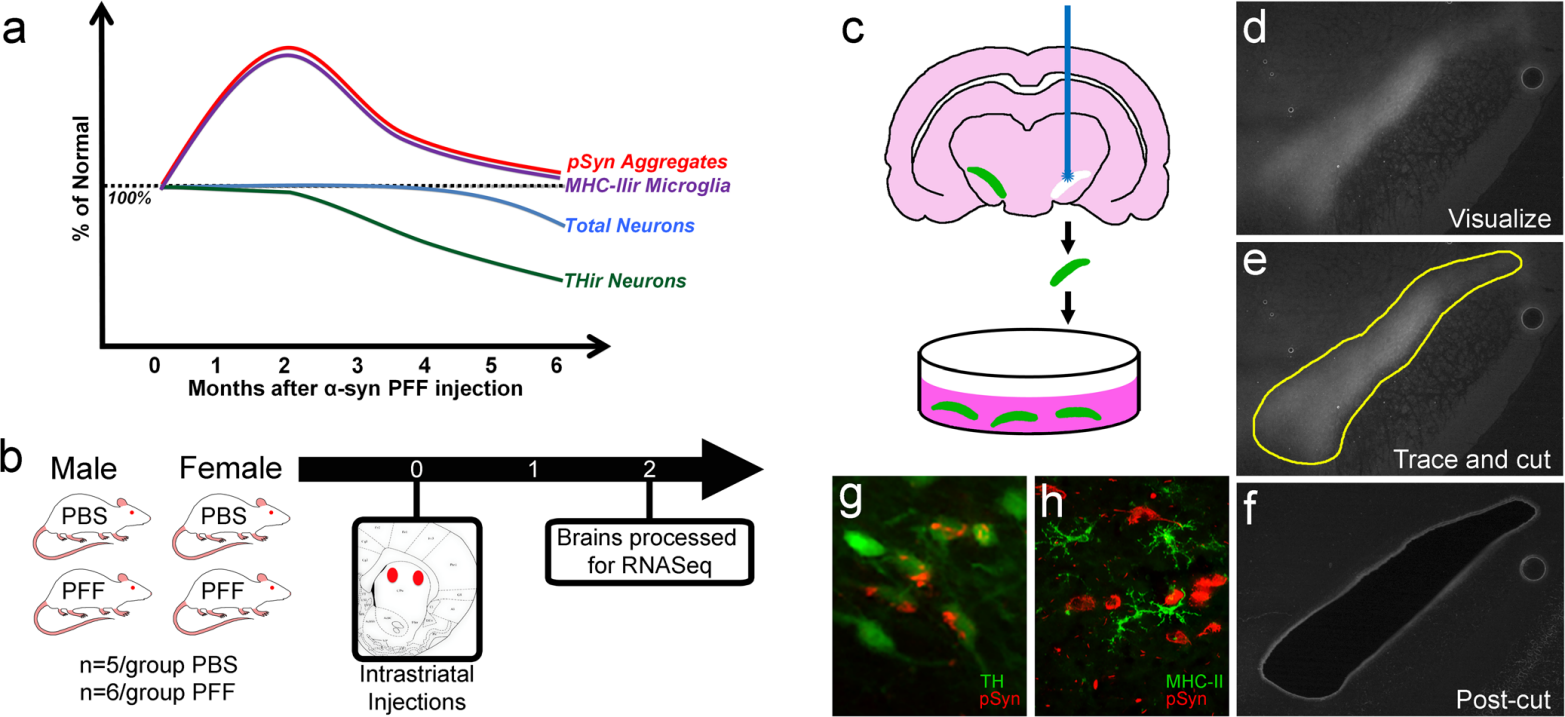
Rat preformed fibril model overview and experimental design. **a** Graphic time course of the features of the preformed fibril (PFF) model in the ipsilateral substantia nigra pars compacta (SNpc), illustrating how the number of phosphorylated alpha-synuclein (pSyn) inclusion containing neurons and major histocompatibility complex II immunoreactive (MHC-IIir) microglia peak at 2 months post-intrastriatal injection of PFF. This is followed by the phenotypic loss of tyrosine hydroxylase immunoreactive (THir) neurons by 4 months, and subsequent total neuron loss by 6 months. The graphic time course synthesizes results from multiple studies using the PFF model in rats^25,27,28,30,68^ and outlines key features as the model progression. **b** Experimental design of the laser capture microdissection (LCM) paired with RNASeq experiment. Male and female hTH-GFP rats received intrastriatal injections of alpha-synuclein preformed fibrils (PFFs) or PBS vehicle as a control condition. At 2 months, brains were removed and processed for LCM-RNASeq. **c** Diagram of the LCM process, showing the SNpc labeled with GFP under the human TH promoter. The SNpc is visualized, traced, cut, and collected in TRIzol for RNA isolation. Micrograph examples of the SNpc during the LCM process, showing **d** the visualization of the SNpc from GFP expression, **e** the borders of the SNpc that are traced, and **f** the appearance of the tissue post-cut, after the dissected tissue has been collected. **g** Micrograph showing pSyn inclusions in dopamine neurons in the SNpc expressing GFP under the human TH promoter. **h** Micrograph showing MHC-II positive microglia in proximity to pSyn inclusions in the SNpc.

Animal models that accurately recapitulate PD pathological hallmarks can help disentangle the complex variables and limitations associated with human PD tissue. The alpha-synuclein (α-syn) preformed fibril (PFF) model demonstrates several key features of idiopathic PD. Intrastriatal injection of PFFs into rodents initiates templating, phosphorylation, and aggregation of endogenous α-syn in neurons within and innervating the striatum^24–30^. These inclusions share multiple features with Lewy bodies in that they contain phosphorylated α-syn (pSyn), ubiquitin, and p62, are positive for amyloid structures based on thioflavin staining, are resistant to proteinase K digestion, and contain fragments of organelles and membranes^24–31^. When PFFs are injected into wildtype mice and rats, these Lewy body-like inclusions form within the context of normal α-syn levels, mirroring the normal α-syn levels associated with idiopathic PD^32^. Both pSyn accumulation and microglial reactivity (based on major histocompatibility complex-II; MHC-II) in the SN pars compacta (SNpc) peak 1-2 months after intrastriatal PFF-injection to rats^27,28^, with MHC-II antigen presentation proportionate to inclusion load^27^ as previously observed in PD tissue^33,34^. Similarly, during peak pSyn accumulation in the SNpc, astrogliosis is increased^30,35–37^ and is positively associated with pSyn accumulation^30^. Following the peak of pSyn accumulation and associated gliosis observed at 2 months, nigrostriatal neurons display significant loss of dopaminergic phenotype (tyrosine hydroxylase immunoreactivity) at 4 months, with subsequent SNpc neurodegeneration occurring between 5-6 months^27,28^ (Fig. 1a).

These synchronized synucleinopathy and neuroinflammatory responses observed in the SNpc in the rodent PFF model at the early 2-month time point provide a unique opportunity to investigate Lewy body-like α-syn inclusion-associated transcriptional alterations that occur in neurons and glia months prior to nigral neurodegeneration. In the present set of experiments, we sought to identify the transcriptomic changes associated with early synucleinopathy and neuroinflammation in the SNpc. PFFs were intrastriatally injected into both male and female transgenic rats expressing EGFP under the human tyrosine hydroxylase (TH) promoter, with EGFP used as the marker to identify and precisely collect the entire SNpc using laser capture microdissection (LCM) followed by RNASeq (Fig. 1b–f). LCM for the specific isolation of the SNpc allowed for collection of both dopaminergic neurons containing pSyn inclusions as well as the proximal glia (Fig. 1g, h). RNA isolation from a larger area would likely dilute the effects and not be specific to nigral dopamine neurons containing inclusions or reactive glia. To confirm and localize transcriptional changes identified via RNASeq, follow-up studies were performed in non-transgenic rats using droplet digital PCR (ddPCR) and fluorescent in situ hybridization (FISH). Within both sexes, we observed that downregulated genes were primarily associated with neurotransmission pathways, specifically synaptic vesicle function, and dopamine handling and release. These results confirm previously reported gene expression changes observed in hippocampal neuronal cultures seeded with pSyn inclusions via PFF exposure^31,38^, while providing additional insight into the specific response of nigral dopamine neurons. Also in both sexes, upregulated genes were largely associated with innate and adaptive immune response pathways, suggesting an early inflammatory response to the presence of inclusion containing nigral neurons prior to degeneration. These results confirm multiple transcriptional changes previously observed in postmortem PD SN, while also identifying new targets for future intervention strategies.

## Results

### Differential transcript expression analysis

**Fig. 2:**
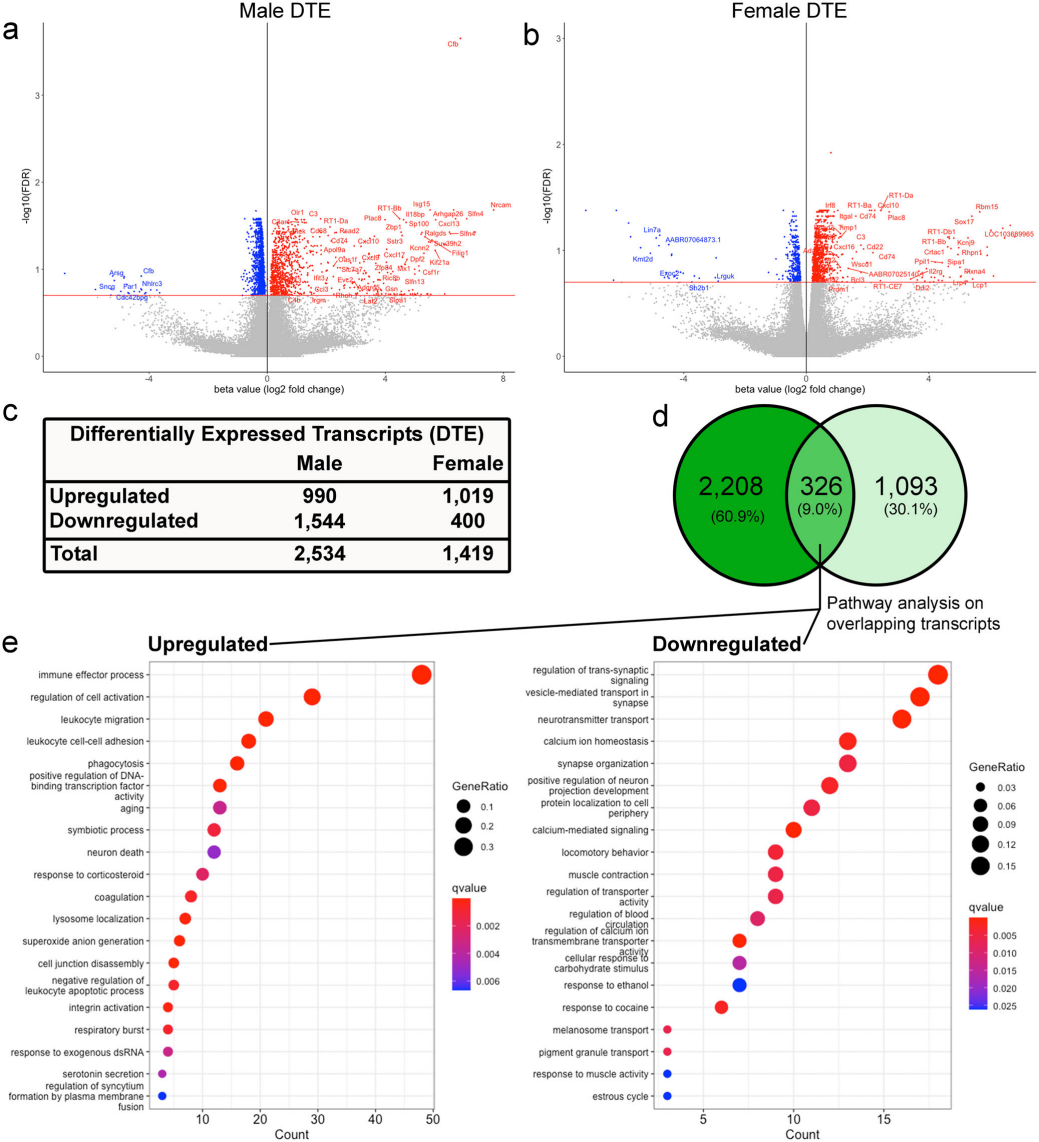
Differential transcript expression (DTE) analysis results. Volcano plots of differential transcript expression (DTE) in **a** males and **b** females with an FDR threshold of <0.2. **c** During testing, we identified 2534 differentially expressed transcripts by PFF treatment in males and 1419 differentially expressed transcripts in females (FDR < 0.2). **d** Venn diagram of differential transcript expression (DTE) results in male and female rats. A total of 326 transcripts were identified as differentially expressed in both sexes. **e** Dotplots of GO term enrichment testing for the up- and downregulated genes that overlapped in males and females. GO term enrichment was performed using a hypergeometric test in the *clusterProfiler* R package. Enriched GO biological process (GOBP) terms for upregulated genes are on the left, enriched GOBP for downregulated genes are on the right. GeneRatio refers to the ratio of genes in the GOBP term to the total number of input genes, and qvalue refers to the Benjamini-Hochberg-adjusted p-value. GeneRatio is represented by the size of dots in the plot, and q-value is represented by color.

Ipsilateral SNpc samples from male and female rats were collected via LCM two months following intrastriatal PFF or PBS injection. In male animals, 495 transcripts displayed a significant change in transcript expression between PFF and PBS treatment (*q* < 0.05, Supplementary Data 1). The stringency of the differential expression testing was then lessened to *q* < 0.2 to include transcripts that could be overlooked due to the small sample size of this discovery study. As such, these transcripts require validation in future studies. When differential expression testing in males was lessened to *q* < 0.2, there were 2534 differentially expressed transcripts (Supplementary Data 1). Of those 2534 differentially expressed transcripts in males, 990 were upregulated and 1544 were downregulated (Fig. 2a, c). In the female rats, 71 transcripts showed a significant difference in PFF-treated rats compared to control (*q* < 0.05, Supplementary Data 1). When stringency was lessened to *q* < 0.2, there were 1419 differentially expressed transcripts (Supplementary Data 1), 1019 of which were upregulated and 400 downregulated (Fig. 2b, c). Due to incomplete annotation of the *Rattus norvegicus* index transcriptome used in analysis, numerous transcripts were not initially annotated during differential testing. From transcripts that met the FDR *q* < 0.2 criteria, there were 1280 and 642 unannotated transcripts in males and females, respectively. The University of California Santa Cruz (UCSC) Genome Browser (RGSC 6.0/rn6) was used to manually annotate these transcripts (noted in Supplementary Data 2). In males, 934 transcripts overlapped a known gene in rats, while 282 transcripts were not linked to a known gene in rats but showed homology in other species, and 64 had no link or homology in other species. In females, 507 transcripts overlapped a known gene in rats, 91 transcripts showed homology in other species, and 44 had no link or homology in other species.

To understand whether similar transcripts showed changes with PFF treatment in males and females, we compared the lists of differentially expressed transcripts in the stratified male and female results. In total, 326 transcripts were significantly changed in both males and females at *q* < 0.2 (Fig. 2d); of those, 172 (52.8%) showed consistent upregulation in both sexes, and 137 (42.0%) showed consistent downregulation (Supplementary Data 2). The remaining 17 overlapping transcripts (5.2%) showed inconsistent patterns of differential expression by sex (Supplementary Data 2). After differential expression testing, we used the *clusterProfiler* package in R to run gene ontology (GO) term and Kyoto Encyclopedia of Genes and Genomes (KEGG) term enrichment testing on the lists of annotated genes that showed consistent upregulation (*n* = 172) and downregulation (*n* = 137) by PFF treatment in both male and female animals. To visualize our results, we generated dotplots of the top 20 enriched GO term pathways for the up/downregulated genes that showed differential expression in both males and females (Fig. 2e, Supplementary Data 3). The most enriched pathway in the upregulated genes was “immune effector process” and the top pathway in the downregulated genes was “regulation of trans-synaptic signaling.”

### Sex-specific gene ontology comparison

To elucidate common pathways in both sexes, our initial GO analysis was performed on differentially expressed transcripts present in both sexes. As a follow-up, we next performed the analysis on genes encoding transcripts differentially expressed in either males or females separately (Supplementary Data 2). Relevant pathways identified by KEGG term enrichment testing in both sexes include: “dopaminergic synapse”, “pathways of neurodegeneration”, “TNF signaling pathway”, “MAPK signaling pathway” and “CAMP signaling pathway” (Supplementary Data 4). Given that more differentially expressed transcripts were observed in males, there were more pathways identified in males than females. Relevant pathways identified in males were: “Parkinson disease”, “synaptic vesicle cycle”, “phagosome”, “circadian entrainment” “circadian rhythm”, “glycolysis/gluconeogenesis”, “chemokine signaling pathway”, “ubiquitin mediated proteolysis”, and others (Supplementary Data 4). Relevant pathways identified in females were: “axon guidance”, “Wnt signaling pathway”, “Fc gamma R-mediated phagocytosis”, and “regulation of the cytoskeleton” (Supplementary Data 4).

### pSyn inclusion comparison between sexes in the SN

Results from the transcriptomic analysis indicate there were more differentially expressed transcripts in males than females. A potential source for this difference could be the efficacy of the PFFs to produce pSyn pathology between sexes. To address this, an additional cohort of both sexes were used to quantify the number of SNpc neurons possessing pSyn at the two-month timepoint. In concordance with the RNASeq results, males had ~32% more pSyn inclusion-containing neurons than females (two-tailed t-test; *p* = 0.0329), with males and females having an average of 3,315.8 ± 348.0 and 2,502.0 ± 143.5 pSyn inclusion-containing neurons in the SNpc, respectively (Supplementary Fig. 1).

### Assessing cell type specificity with DropViz database

To further refine the results, data were cross-referenced with the DropViz database (http://dropviz.org) to sort transcripts based on expression in known cell types. As DropViz is a mouse database, rat gene names were converted to mouse gene names using the *biomaRt* R package. We first analyzed transcripts known to be expressed in SN neurons, specifically *Anxa1*, *Grin2c*, *Vcan*, and *Cyp26b1. Anxa1*, *Grin2c*, and *Vcan* are markers for the Aldh1a1-positive subtype of DaNs in the ventral SNpc^39–42^, which are particularly susceptible in PD^43^. *Cyp26b1* is a marker for dopamine neurons in the compacta which do not express Aldh1a1^39–42^. In our RNAseq data, 12,735 transcripts in males and 12,802 transcripts in females are present in cells also expressing *Anxa1*, *Grin2c*, *Vcan*, and *Cyp26b1* mRNA in the SN via the DropViz database (Supplementary Data 5, Fig. 3a, b). From these transcripts, 998 in males and 654 in females respectively were differentially expressed (*q* < 0.2). Inclusion of *Cyp26b1* only added *Casp4* to the list of transcripts present in nigral dopamine neurons. The relevant GO and KEGG term associated with the upregulated transcripts in both sexes was “immune effector process”, whereas downregulated transcripts were associated with “regulation of vesicle-mediated transport”, “regulation of trans-synaptic signaling”, “dopamine synapse”, “synapse organization,” “locomotory behavior”, “calcium ion homeostasis”, and “calcium-mediated signaling” (Supplementary Data 6).

**Fig. 3:**
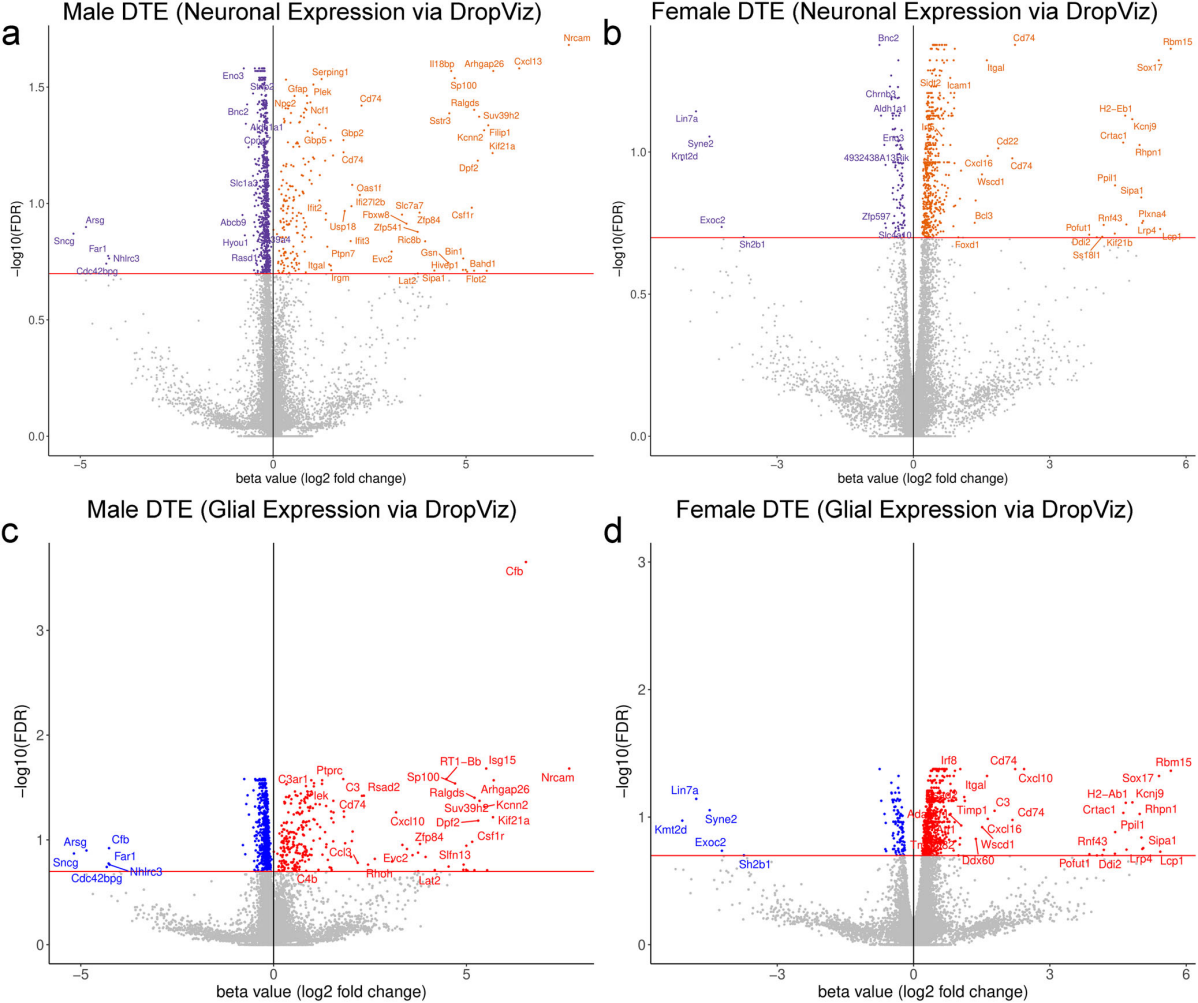
Differential transcript expression results segregated by sex and predicted cell type based on DropViz. Differential transcript expression results were separated by sex, then organized by transcripts present in dopamine neurons or glia based on the DropViz database. Transcripts present in *Anxa1*, *Grin2c*, *Vcan*, and *Cyp26b1* positive cells in the SN in the DropViz database were considered to be present in nigral dopamine neurons. Transcripts present in *Myoc*, *Cst3*, *Igfbp2*, and *Tmem119* positive cells in the DropViz database were considered to be present in microglia and astrocytes. Neuronal expression in **a** males, **b** females, and glial expression in **c** males and **d** females are expressed as volcano plots with an FDR threshold set at <0.2.

To consider other cell types of interest, *Myoc*, *Cst3*, and *Igfbp2* were used as transcriptional markers for astrocytes and *Tmem119* mRNA expression as a marker for microglia. There were 12,870 transcripts in males and 12,946 transcripts in females, with 1,043 in males and 689 in females differentially expressed (*q* < 0.2) in our RNASeq data in either astrocytes or microglia (Supplementary Data 5, Fig. 3c, d). The relevant GO and KEGG terms associated with the upregulated transcripts were “immune effector process”, “regulation of cytokine production”, and “complement and coagulation cascades,” whereas downregulated transcripts were associated with “signaling transduction pathways” (Supplementary Data 7).

### Weighted gene correlation network analysis

In addition to differential expression testing, we performed Weighted Gene Correlation Network Analysis (WGCNA), an unsupervised clustering algorithm that groups genes into arbitrary modules based on correlated expression values. To limit the scope, modules were filtered down to “hub” genes, or those with the top 10% greatest module membership score in each module^44^. This analysis was separate from the differential expression testing and was performed to further narrow our results to key regulators that may be involved in early synucleinopathy.

In total, 22 transcripts were identified as differentially expressed hub genes in both sexes (Supplementary Table 1). Of these, 12 (54.5%) showed consistent upregulation by PFF treatment in both sexes, 5 (22.7%) showed consistent downregulation by PFF treatment in both sexes, and 5 (22.7%) showed inconsistent direction between sexes. Initially, 5 of the 22 hub genes were not annotated to a known gene based on the *Rattus norvegicus* reference index. As such, these genes were manually annotated via the UCSC genome browser (denoted in bold and italicized font in Supplementary Table 1). All annotated hub genes were considered potential key drivers of the effects of early synucleinopathy and were input into our pathway and network analyses. Similar to the differential transcript expression analysis testing, upregulated hub genes showed enrichment for immune-related pathways including “immune effector process” and “microglial cell activation,” while downregulated hub genes showed enrichment for dopamine signaling pathways, including “dopamine transport” and “regulation of dopamine receptor signaling pathway.”

### Validation of the effects of early synucleinopathy on genes involved in neurotransmission

**Fig. 4:**
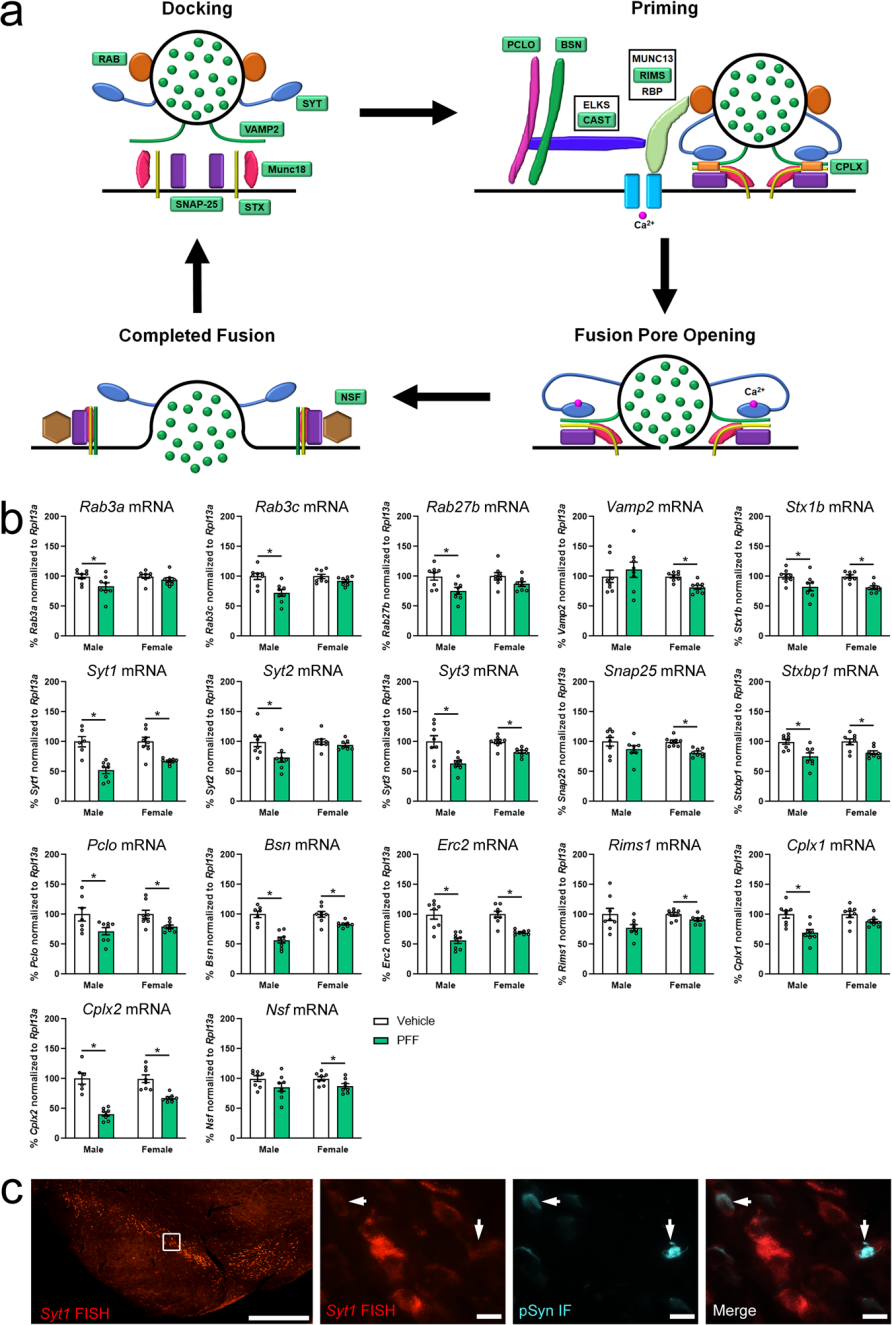
Downregulation of general neurotransmission-related genes in early synucleinopathy. **a** Diagram of the vesicular release process during neurotransmission, showing the docking, priming, fusion pore opening, and fusion stages. Proteins encoded by transcripts downregulated in early synucleinopathy are highlighted in green. **b** Fischer 344 rats received two intrastriatal injections of 4 µg/µL α-syn PFFs or PBS (Vehicle), SN was collected 2-months after PFF administration from flash-frozen tissue and processed for ddPCR. Graphs show mRNA from genes in the general vesicular release process normalized to *Rpl13a* mRNA and measured via ddPCR. Transcripts assessed were *Rab3a* (Ras-associated binding protein 3a), *Rab3c* (Ras-associated binding protein 3c), *Rab27b* (Ras-associated binding protein 27b), *Vamp2* (synaptobrevin 2), *Stx1b* (syntaxin 1b), *Syt1* (synaptotagmin 1), *Syt2* (synaptotagmin 2), *Syt3* (synaptotagmin 3), *Snap25* (synaptosome associated protein 25), *Stxbp1* (syntaxin binding protein 1, also known as MUNC18), *Pclo* (piccolo), *Bsn* (bassoon), *Erc2* (ELKS/RAB6-interacting/CAST family member 2, also known as CAST1), *Rims1* (regulating synaptic membrane exocytosis 1), *Cplx1* (complexin 1), *Cplx2* (complexin 2), and *Nsf* (N-ethylmaleimide sensitive factor, also known as vesicular fusion protein NSF). Columns indicate the group means, circles represent individual data points (*n* = 8 per group before outlier removal), and error bars represent ±1 standard error of the mean. An asterisk represents significance (*p* ≤ 0.05). Outliers were removed based on the absolute deviation from the median method. **c** Male Fischer 344 rats received two intrastriatal injections of 4 µg/µL α-syn PFFs, brains were removed 2-months after PFF administration and processed for fluorescent in situ hybridization (FISH). Panels show representative *Syt1* FISH micrographs from the SNpc White box denotes the area of the higher magnification images, which show *Syt1*mRNA (red) and immunofluorescent staining for pSyn (green). White arrows denote neurons containing pSyn inclusions which show a qualitative decrease in *Syt1* mRNA. Scale bars are 500 µm in the lower magnification image, and 10 µm in the higher magnification images.

RNASeq results in males and females at 2 months post-PFF demonstrated the downregulation of several genes encoding proteins critical to the general neurotransmission/vesicular release process (Fig. 4a). Since RNASeq results were from transgenic hTH-EGFP rats and the accuracy of transcript alignment was dependent on the reference genome, ddPCR with transcript specific primer/probes was used with a separate validation cohort of non-transgenic male and female Fischer 344 rats to confirm a subset of the RNASeq results. We assessed transcripts that were downregulated in at least one sex in RNASeq, while also extending the analysis to include other related genes. Compared to our RNASeq results, the ddPCR experiments showed similar downregulation of neurotransmission related transcripts, but there were some discrepancies based on sex (Supplementary Table 2). Where we saw *Syt1*, *Snap25*, *Pclo*, *Erc2*, *Rab3c*, and *Cplx2* transcripts decreased at 2 months post-PFF in both sexes with RNASeq, only *Syt1*, *Pclo*, *Erc2*, and *Cplx2* were decreased in both sexes with ddPCR (Fig. 4b; Supplementary Data 8). It is important to note that though *Pclo* and *Erc2* decreased in both sexes in the RNASeq, these decreases were observed in different transcribed regions of the mRNA (marked as separate transcripts in the RNASeq), which is why they are not present in our list of 326 differentially expressed transcripts (Supplementary Data 2). In contrast to the RNASeq data in both sexes, *Snap25* only decreased in females and *Rab3c* only in males 2 months post-PFF in the ddPCR follow-up study (Fig. 4b; Supplementary Data 8). In the RNASeq results, *Vamp2*, *Rims1*, *Bsn*, *Cplx1*, *Rab3a*, *Rab27b*, *Stxbp1*, *Nsf*, and *Stx1b* only decreased in males at 2 months post-PFF, which remained the same for *Cplx1*, *Rab3a*, and *Rab27b* when assessed with ddPCR (Fig. 4b; Supplementary Data 8). In contrast to the RNASeq data which showed decreases only in males, *Bsn Stxbp1*, and *Stx1b* decreased in both sexes, whereas *Vamp2*, *Rims1*, and *Nsf* only decreased in females at 2 months post-PFF (Fig. 4b; Supplementary Data 8). Additionally, we used ddPCR to examine two other members of the synaptotagmin (*Syt)* family: *Syt2* and *Syt3*. *Syt2* was only decreased in males and *Syt3* was decreased in both sexes (Fig. 4b; Supplementary Data 8).

**Fig. 5:**
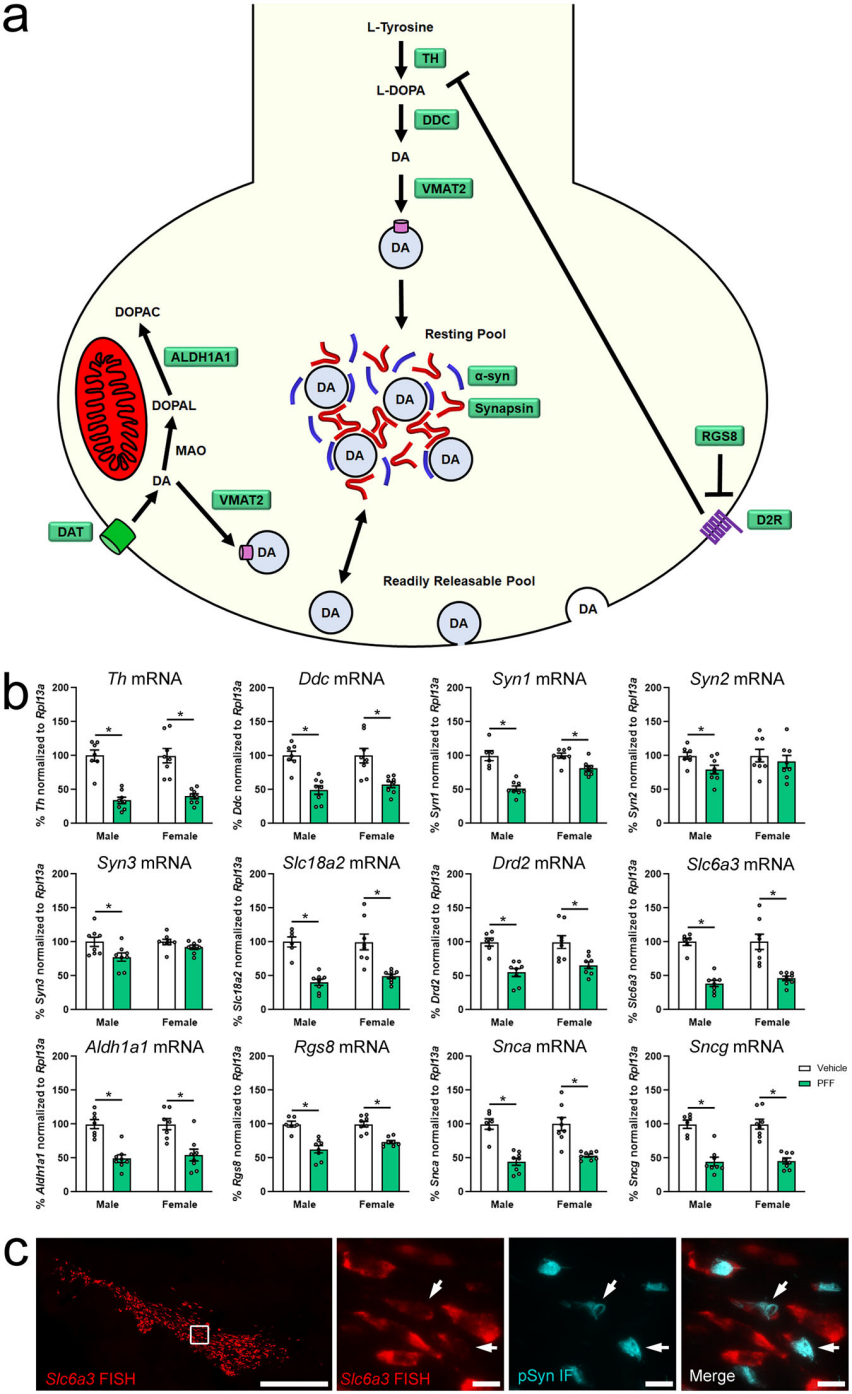
Downregulation of dopamine pathway related genes in early synucleinopathy. **a** Diagram of the dopamine pathway and vesicle organization via synapsins and α-syn. Proteins encoded by transcripts downregulated in early synucleinopathy are highlighted in green. **b** Fischer 344 rats received two intrastriatal injections of 4 µg/µL α-syn PFFs or PBS (Vehicle), SN was collected 2-months after PFF administration from flash frozen tissue and processed for ddPCR. Graphs show mRNA from genes in the dopamine pathway normalized to *Rpl13a* mRNA and measured via ddPCR. Transcripts assessed were *Th* (tyrosine hydroxylase), *Ddc* (DOPA decarboxylase), *Syn1* (synapsin 1), *Syn2* (synapsin 2), *Syn3* (synapsin 3), *Slc18a2* (vesicular monoamine transporter 2 or VMAT2), *Drd2* (dopamine receptor D2), *Slc6a3* (dopamine transporter or DAT), *Rgs8* (regulator of G protein signaling), *Aldh1a1* (aldehyde dehydrogenase 1 family member A1), *Snca* (alpha-synuclein or α-syn), and *Sncg* (gamma-synuclein or γ-syn). Columns indicate the group means, circles represent individual data points (*n* = 8 per group before outlier removal), error bars represent ±1 standard error of the mean. An asterisk represents significance (*p* ≤ 0.05). Outliers were removed based on the absolute deviation from the median method. **c** Male Fischer 344 rats received two intrastriatal injections of 4 µg/µL α-syn PFFs, brains were removed 2-months after PFF administration and processed for fluorescent in situ hybridization (FISH). Panels show representative FISH micrographs from the SNpc dopamine pathway specific transcript *Slc6a3*. White box denotes the area of the higher magnification images, which show *Slc6a3* mRNA (red) and immunofluorescent staining for pSyn (cyan). White arrows denote neurons containing pSyn inclusions which show a qualitative decrease in *Slc6a3* mRNA.

Similar to general neurotransmission-associated genes, several dopamine pathway and vesicle trafficking-related transcripts were also downregulated (Fig. 5a). We showed a decrease at 2 months post-PFF in *Ddc*, *Slc18a2*, *Drd2*, *Rgs8*, *Slc6a3*, and *Aldh1a1* in both sexes (Supplementary Data 2), and the validation cohort using ddPCR likewise showed these transcripts decreased at 2 months post-PFF in both sexes (Fig. 5b; Supplementary Data 8). One transcript which only decreased in males in the RNASeq results was *Th*, which in contrast was decreased in both sexes at 2 months post-PFF in the ddPCR study (Fig. 5b; Supplementary Data 8).

In addition to the dopamine pathway, we also identified treatment-associated downregulation of transcripts encoding synapsins and synucleins, which are predicted to be involved in vesicle organization and trafficking. We showed that *Syn2* and *Syn3* decreased only in males at 2 months post-PFF by RNASeq (Supplementary Data 2). Further, we replicate this finding in the ddPCR study at 2 months post-PFF (Fig. 5b; Supplementary Data 8). Based on these results, we extended our assessment to *Syn1*, which was not implicated in the RNASeq data, but was decreased at 2 months post-PFF in both sexes by ddPCR (Fig. 5b; Supplementary Data 8). Using RNASeq, we saw decreased *Snca* transcript in both sexes and decreased *Sncg* in only males at 2 months post-PFF (Supplementary Data 2). Similar to Pclo and *Erc2*, *Snca* decreases were observed in different transcribed regions of the mRNA (marked as separate transcripts in the RNASeq), which explains why *Snca* is not present in list of 326 differentially expressed transcripts (Supplementary Data 2). Examination of *Snca* and *Sncg* with ddPCR showed a decrease in *Snca* in both sexes, thereby validating the RNASeq results (Fig. 5b; Supplementary Data 8). However, in contrast to the RNASeq results, the decrease in *Sncg* was not limited to males, but was present in both sexes at 2 months post-PFF in the ddPCR validation study (Fig. 5b; Supplementary Data 8).

Taken together, RNASeq and targeted confirmatory ddPCR identified consistent downregulation in the SNpc in response to early synucleinopathy and validated the downregulation of WGCNA identified “hub genes” *Cplx2, Slc6a3*, and *Rgs8*. However, these methods did not determine the cells in the SNpc where these transcriptional changes occur. To localize transcriptional changes in the SNpc, we used FISH to examine *Syt1* from the general neurotransmission-related transcripts, and *Slc6a3* from the dopamine pathway. *Syt1* (Fig. 4c) and *Slc6a3* (Fig. 5c) transcript expression both roughly mirror the neuronal expression pattern that would be expected of tyrosine hydroxylase immunoreactivity in the nigrostriatal dopamine neurons in the SNpc. Dual labeling with FISH and immunofluorescence for pSyn showed a qualitative decrease in *Syt1* (Fig. 4c) and *Slc6a3* (Fig. 5c) in pSyn-containing neurons compared to neighboring neurons. These data suggest that the degree of loss of neurotransmission and dopamine pathway-related transcripts observed in the dissected tissue may be driven by changes only occurring in pSyn inclusion-containing neurons, rather than all or pSyn inclusion-adjacent nigrostriatal dopamine neurons. If this is the case, then it is likely single-cell analysis of pSyn inclusion containing neurons would show an even greater degree of differential transcript expression than analysis from the entire SNpc.

## Discussion

In this study we leveraged the peak accumulation of pSyn inclusions in the SNpc, observed two months following intrastriatal PFF injections in rats and months prior to neurodegeneration^27,28^, to identify early differential gene expression in synucleinopathy.

Through the use of LCM guided by GFP under the hTH promoter, we were able to isolate and collect the SNpc from samples, providing enrichment of RNA from nigral dopamine neurons that possess pSyn inclusions as well as immediately proximal glia. Overall, transcripts downregulated in early synucleinopathy in both sexes were primarily associated with neurotransmission and the dopamine pathways, whereas those upregulated in early synucleinopathy were primarily associated with the neuroinflammatory response.

Neurotransmitter release is a choreographed process that requires the interaction of numerous proteins. Both RNASeq and the ddPCR results in early synucleinopathy show the downregulation of several transcripts encoding protein involved in vesicle organization and neurotransmitter release, such as *Syn1* (synapsin 1), *Syn2* (synapsin 2), *Syn3* (synapsin 3), *Vamp2* (synaptobrevin 2), *Snap25* (SNAP25), *Stx1b* (syntaxin 1b), *Stxbp1* (Stxbp1 also known as MUNC18), *Pclo* (piccolo), *Bsn* (bassoon), *Erc2* (CAST1), *Rims1* (Rims1), *Rab3a* (RAB3a), *Rab3c* (RAB3c), *Rab27b* (RAB27b), *Syt1* (synaptotagmin 1), *Syt2* (synaptotagmin 2), *Syt3* (synaptotagmin 3), *Cplx1* (complexin 1), *Cplx2* (complexin 2), and *Nsf* (N-ethylmaleimide sensitive factor, vesicle fusing ATPase). Synaptic vesicles in the axon terminals have been hypothesized to be organized and trafficked into three pools prior to neurotransmitter release: the resting or reserve pool, the recycling pool, and the readily releasable pool. The reserve pool is organized by synapsin proteins interacting with α-syn and has been suggested to be the largest pool of SVs that can feed into the recycling pool if needed^45–47^. The recycling pool is comprised of SVs that can move into the readily releasable pool when needed, and the readily releasable pool is comprised of synaptic vesicles that are found in the active zone^45–52^. Decreases in Syn*1* have been reported in α-syn overexpression in culture^53^, rodent models^54,55^, cultured mouse hippocampal neurons treated with PFFs^31^, as well as in post-mortem tissue from patients with dementia with Lewy bodies (DLB)^53^. Similarly, Syn*2* decreases in α-syn overexpressing mice^55^, cultured mouse hippocampal neurons treated with PFFs^38^, and post-mortem tissue from PD patients^13^. Reported changes in Syn*3* are variable, with increases reported in post-mortem PD tissue^55^, and decreases reported in iPSCs harboring the A53T *SNCA* mutation^56^. Downregulation of synapsins in early synucleinopathy suggests disorganization of synaptic vesicles, likely delaying vesicle movement to the active zone and impairing dopamine release in the nigrostriatal pathway.

During the neurotransmitter release process, synaptic vesicles in the active zone dock and prime for release via interactions between vesicular-SNARE protein synaptobrevin, and the target-SNARE proteins SNAP-25, syntaxin, and Munc18 on the synaptic membrane^48–52^. The complexin proteins bind to the formed SNARE complex to regulate fusion between the vesicle and cell membrane^48–52^. Priming of synaptic vesicles is also modulated by the interaction of Rab proteins and the presynaptic cytoskeletal matrix of the active zone, which is comprised of scaffolding proteins such as piccolo, bassoon, CAST, ELKS, Munc13 and the RIMS proteins^48–52^. Together, these are predicted to aid in maintaining proximity to calcium channels^48–52^. Stimulation of the neuron to fire results in the binding of calcium with synaptotagmin on the synaptic vesicle, which displaces complexin and results in membrane fusion and release of the neurotransmitters^48–52^. Regarding components of the SNARE complex, syntaxin 1 is decreased in post-mortem tissue from DLB patients^57^, as well as synuclein overexpression models in mice^55^ and rats^58^. Changes in synaptobrevin 2 vary by method, with decreases observed in hippocampal cultures overexpressing α-syn or with intracellular PFF-induced inclusions^38,53^, and increases in α-syn overexpressing mice^55^. SNAP-25 protein is also variable, with decreases reported in post-mortem DLB and PD with dementia patients^59,60^ and cultured mouse hippocampal neurons treated with PFFs^38^; and increases in α-syn overexpressing in mice^55^. Complexins 1 and 2 are decreased in α-syn overexpressing mice^55^, with complexin 2 also decreased in post-mortem tissue from PD patients^55^. Involving priming, decreases in Rab3A have been reported in post-mortem tissue from PDD and DLB patients^59,60^, as well as synuclein overexpression models in mice^55^ and rats^58^. Decreases in the cytomatrix scaffold protein piccolo in cultured mouse hippocampal neurons overexpressing a-syn have also been reported^53^. We observed decreases in synapsins and synaptobrevins, and the complexins and piccolo transcripts generally mirror previous findings associated with protein changes; however, this does not hold true for synaptotagmin. Where we see a decrease in *Syt* transcripts, synaptotagmin 1 is increased in α-syn overexpressing mice^55^, and synaptotagmin 2 is increased in post-mortem and priming in the active zone based on deficits in the SNARE complex and cytomatrix proteins, and impaired membrane fusion and fusion pore opening due to decrease synaptotagmin. These biological changes would be expected to decrease dopamine release in the nigrostriatal pathway in early synucleinopathy and potentially produce stress in the axon terminals that could contribute to axonopathy.

Previous studies revealing dysregulation of neurotransmission transcripts and proteins associated with PFF-induced pSyn inclusions used hippocampal cultures^31,38^. However, nigral dopamine neurons have unique firing patterns^61^ and use proteins involved in dopamine synthesis and handling that hippocampal cultured cells do not express. In DaNs, dopamine is synthesized in a stepwise process where L-tyrosine is converted to L-DOPA by tyrosine hydroxylase (TH), and DOPA decarboxylase (DDC) converts L-DOPA to dopamine. Models employing α-syn overexpression show decreased *Th* and *Ddc* transcripts^62^ and parallel reduction in TH protein/activity^63–67^ and DDC protein^67^. Similarly, we observed decreases in *Th* and *Ddc* transcript levels in early synucleinopathy. However, previous findings show that TH immunoreactivity and dopamine synthesis and storage in the nigrostriatal system remains relatively unchanged until 4 months after PFF injection to rats^28,68^, suggesting a delay between *Th* transcript and protein decreases.

After synthesis, dopamine is packaged into synaptic vesicles for release via vesicular monoamine transporter 2 (VMAT2)^69,70^. Dopamine released in the synapse can interact with post-synaptic dopamine receptors on medium spiny neurons, presynaptic dopamine receptors on the axon terminal, or can be transported back into the presynaptic terminal via the dopamine transporter (DAT). Presynaptic dopamine neurons express the inhibitory subtype D2 dopamine receptor subtype (D2R) on the presynaptic terminal. D2R is a G-protein coupled receptor which ultimately decreases TH activity to reduce dopamine synthesis^71^. The activity of G-protein coupled receptors are modulated by the regulator of G-protein signaling (RGS) proteins, with RGS8 being a known regulator of the D2R activity^72,73^. In the present analysis, both *Drd2* and *Rgs8* transcripts decrease in early synucleinopathy. As D2R inhibits TH activity, it is possible that downregulation of *Drd2* is a compensatory mechanism to promote dopamine synthesis. Decreased *Rgs8* transcript however is contradictory to the compensatory dopamine synthesis hypothesis, as the decrease in RGS8 protein would likely promote D2R-related inhibition of TH activity, since RGS8 normally functions by increasing the rate of D2R inactivation.

Our RNAseq analysis identified decreased transcription of the monoamine transporters *Slc18a2* (VMAT2) and *Slc6a3* (DAT), in early synucleinopathy. At the 2-month post-PFF time point, previous work shows that decreased DAT function in the striatum is already present^68^, suggesting transcriptional changes translate to functional losses. Similar to our findings, overexpression of α-syn in culture results in inhibition of VMAT2 activity and decreased DAT protein and function^74–80^. As the primary function of the nigrostriatal dopamine neurons is to synthesize and release dopamine – thereby maintaining balance in the basal ganglia––downregulation of these genes can negatively affect the overall function of these neurons. Decreases in TH and DDC protein would reduce dopamine synthesis, where decreases in VMAT2 and DAT protein would negatively affect dopamine packaging into vesicles for release and reuptake/reuse of dopamine, respectively. Impairment in the dopamine pathway along with deficits in synaptic vesicle organization and general neurotransmission machinery likely produce synaptic dysfunction and thus, may contribute to the nigrostriatal axonopathy observed in early PD^81,82^.

In addition to dopamine release and reuptake, dopamine metabolism also has the potential to influence the health of the neurons. Dopamine can be metabolized by monoamine oxidase to produce DOPAL, which can be converted to DOPAC by ALDH1A1^83,84^. In our results, *Aldh1a1* transcript decreased early in synucleinopathy. Inhibition or dysfunction of ALDHs lead to the accumulation of DOPAL, a toxic metabolite of dopamine. DOPAL can act by inhibiting TH activity^85^, producing toxic quinones resulting in ROS^86^, and modifying and promoting α-syn oligomerization which can impair synaptic vesicle function^87–95^. The resulting synaptic distress can decrease dopamine release, leading to axonopathy, and ultimately, neurodegeneration. Deficiencies in ALDH1A1 have previously been associated with PD, and the subtype of dopamine neurons which produce ALDH1A1 show increased vulnerability in PD^43^. Downregulation of both *ALDH1A1* mRNA^17,96^ and ALDH1A1 protein^97,98^ have been observed in post-mortem PD tissue from patients with both familial and idiopathic PD^97,98^. Related to the loss of ALDH1A1, DOPAL accumulation in the putamen has been found in post-mortem PD tissue^99^. Given the downregulation of *Aldh1a1* that we observe in early synucleinopathy and potential loss of a protective enzyme, DOPAL accumulation and toxicity are mechanisms which could contribute to synucleinopathy progression and should be examined in future studies.

Along with the differential expression of several genes related to neurotransmission and the dopamine pathway, we showed that synuclein transcripts *Snca* and *Sncg* decrease in early synucleinopathy induced via PFFs. This is in stark contrast to what would be expected in α-syn overexpression models and familial forms of PD, such as *SNCA* duplication and triplication^100^. However, previous work has shown decreases in *SNCA* transcript in LCM-dissected post-mortem PD vs control nigral neurons^23^, as well as in primary mouse hippocampal neurons with PFF-induced α-syn inclusions^31^. The mechanism whereby *Snca* and *Sncg* are downregulated in inclusion-bearing neurons remains unknown; however, it is possible that formation of inclusions affects transcriptional regulators controlling *Snca* and *Sncg*.

Nigral samples in our study included multiple cell types immediately adjacent to α-syn inclusion bearing nigral dopamine neurons, including microglia and astrocytes. We found that upregulated transcripts were primarily associated with the neuroinflammatory response, suggesting that the immune system may be triggered by early α-syn pathology. The immune pathways upregulated (Dot plot GO enriched pathway census; (Fig. 2e)) included immune effector processes, cytokine production, B cell activation, and leukocyte activation. Cross-referencing via DropViz (Supplementary Data 7) to sort known glial transcripts further identified the upregulation of pathways involving the complement system, astrocyte function, antigen presentation, and phagocytosis. Many of these upregulated inflammatory pathways mirror findings from the SN of early Braak stages 1 and 2 PD subjects^13^, including upregulation of MHC-II and increased presence of B cells. Furthermore, elevation of complement system components has been detected in postmortem PD tissue^101,102^. Increased microglial MHC-II, astrocytic glial fibrillary acidic protein (GFAP) and complement expression have previously been detected in the pSyn-bearing SN following PFF injection using immunohistochemical methods^27,30,35,103,104^. While these neuroinflammatory results are intriguing, additional experiments are needed to determine the cellular source(s) (e.g. microglia vs. astrocytes vs. other cell types) of the upregulated immune transcripts in our dataset. Further, the neuroinflammatory transcriptome we observe is likely PFF model stage specific^105^, as has been reported in other disease models^106^. Future studies will be required to determine whether the neuroinflammatory transcriptome in the SN during established pSyn aggregation is distinctive from the transcriptome observed during initial aggregate accumulation or during/following nigral degeneration. Additionally, future studies should examine whether the neuroinflammatory transcriptome we observe was impacted by cross-species immune involvement (mouse α-syn injected into rat). Overall, our findings illustrate the complex heterogeneity of the immune system response to accumulation of α-syn aggregation in the SN and suggest that identification of critical drivers of neuroinflammation will be required to develop efficacious anti-inflammatory therapeutic strategies.

Another point to consider are the sex differences that we observed in our results. There is a greater incidence of PD, approximately two-fold higher, in males than females^107,108^; however, females with PD progress faster and have a higher mortality rate^108,109^. We unexpectedly observed a significant difference between male and female rats in the number of pSyn immunoreactive neurons in the SNpc at the 2-month time point: ~32% more pSyn inclusion-containing neurons were observed in the SNpc in males compared to females. We also observed over twice as many differentially expressed transcripts in PFF males than PFF females. These data suggest that the differences in pSyn pathology may underlie transcriptomic differences, but it is also possible that sex-specific differences in the transcriptomic response to PFFs influence pathology. Gene ontology using sex-specific differential transcript expression identified additional pathways that did not emerge when consensus differentially expressed genes (both sexes) were examined. The top pathway identified in males was “Parkinson’s disease”. Enriched pathways identified in males which would be expected to be related to PD are pathways involved in bioenergetics and the mitochondria such as “carbon metabolism”, “glycolysis/gluconeogenesis”, “citrate cycle (TCA cycle)”, and “pyruvate metabolism”; pathways involved in response to protein aggregates such as “lysosome” and “ubiquitin mediated proteolysis”; and pathways involved in sleep disturbances “circadian rhythm” “circadian entrainment”. An example of a pathway implicated in females but not males was the “Wnt signaling pathway”, which interestingly shows sex-specific alterations in female post-mortem PD tissue^12^. Taken together, our results identified sex-specific changes in transcription during early synucleinopathy, but future studies are required to delineate true sex-specific differential transcript expression associated with α-syn inclusions in the SN in which equivalent pSyn deposition is achieved.

It is important to note the limitations of our approach. First, our refined bulk RNAseq approach does not allow for disambiguation of gene expression changes between neuronal or glial subtypes. However, by cross-referencing our transcriptomic data with the DropViz SN neuron and glial databases, as well as direct cell-specific confirmation of mRNA expression using FISH, we have begun to identify markers that can be used in future single-cell RNASeq studies to specifically identify inclusion-bearing nigral neurons in animal models and PD SNpc. Second, our study is limited to understanding the effects of synucleinopathy on the nigral transcriptome, meaning the impact of synucleinopathy on protein expression in the SNpc or striatum remains to be determined. A third limitation is the poor annotation of the rat genome and overall lack of genetic tools in rats. For example, ~50% of the differentially expressed transcripts lacked annotation in males and ~45% in females. Even after manual annotation, ~14% of differentially expressed transcripts in males and 9% in females could not be annotated to a known gene in the rat genome. This incomplete annotation likely resulted in unmatched gene names and gaps in the data. Poor genome annotation could also contribute to misalignment/misidentification of transcripts. For example, *Eno3*, an enzyme in glycolysis/gluconeogenesis, was one of the DTEs identified via RNASeq; however, *Eno3* is primarily expressed in skeletal muscle and was below detectable limits in the SN with ddPCR (data not shown), whereas *Eno2* is expressed in the brain^110–112^. This emphasizes the need to validate RNASeq data, especially in rats. Another limitation of our approach is that PBS and not mouse α-syn monomer was used as the control injectate. Whereas PBS injections control for the effects of intrastriatal surgical injection, we cannot rule out the some of the transcriptional changes we observed following PFF injection may be due to presence of a foreign protein, or due to elevated α-syn. Future studies should include intrastriatal injections of an equal quantity of α-syn monomer for comparison. If properly prepared^113^, injection of α-syn monomer does not result in aggregate formation.

In conclusion, using our approach to enrich collection of α-syn inclusion bearing nigral tissue, we have identified large-scale changes in the transcriptome and multiple pathways altered in early synucleinopathy. Given that the differential transcript expression that we observe occurs months prior to neurodegeneration, the dataset that we have generated can provide insight into the underlying mechanisms of synucleinopathy progression. If validated in PD SN, targets initially identified in the rat PFF model could guide development of future disease-modifying therapies.

## Methods

### Animals (RNASeq)

Male and female 3-month-old hTH-EGFP rats (*n* = 5 PBS groups/sex and *n* = 6 PFF groups/sex) were obtained from Taconic Biosciences (Taconic #12141; NTac:SD-Tg (TH-EGFP) 24Xen). Rats carry an EGFP transgene driven by the human TH promoter inserted on the X chromosome. A breeding colony was established to produce heterozygous females and hemizygous male hTH-EGFP to be used with LCM. Rats were housed 2-3 to a cage in a room with a 12 h light/dark cycle and provided food and water *ad libitum*. All animal work was performed in the Michigan State University Grand Rapids Research Center vivarium. All procedures were approved and conducted in accordance with the Institute for Animal Care and Use Committee (IACUC) at Michigan State University, and the Animal Care and Use Review Office (ACURO) of the United States Army Medical Research and Development Command Office of Research Protections.

### Animals (ddPCR validation and pSyn/FISH assessment in the SN)

Three-month-old male and female Fischer 344 rats were purchased from Charles River Laboratories. For the ddPCR validation study, 8 per sex/group (total *n* = 32) were used. For assessment of pSyn inclusions in the SN and FISH, 8 males per group and 10 females per group were used (total *n* = 36). Rats were housed 1-3 per cage in a room on 12 h light/dark cycle, and food and water were provided *ad libitum*. In all cases where an animal was single housed, enrichment was provided. All animal work was performed in the Michigan State University Grand Rapids Michigan State Research Center vivarium. All procedures were approved and conducted in accordance with the Michigan State University Institute for Animal Care and Use Committee (IACUC) at Michigan State University.

### α-syn PFF preparation

PFFs were generated in the laboratory of Dr. Kelvin Luk at the University of Pennsylvania from recombinant monomeric mouse α-syn and assessed for quality as previously described^24,38,113–115^. PFFs were diluted to 4 µg/µl in sterile Dulbecco’s PBS (PBS) and sonicated at room temperature using an ultrasonic homogenizer (Q125 Sonicator; Qsonica, Newtown, CT); amplitude 30%, 60, 1 s pulses with 1 s between pulses^114^. An aliquot of sonicated PFFs was prepared and analyzed via transmission electron microscopy. Prior to surgery, 20 representative fibrils were measured to ensure fibril length was approximately 50 nm, the reported optimal fibril length for seeding^116,117^. After surgery, over 500 fibrils were measured to calculate the average fibril length and length distribution. All measurements of PFFs were performed using ImageJ^118^. Fibril length distribution ranged from 18.5 to 136.5 nm, with an average size of 47.9 ± 0.7 nm and 81.6% of sonicated PFFs measured ≤60 nm (Supplementary Fig. 2).

### Stereotaxic Surgeries

Male and female rats received a total of 16 µg of PFFs (4 μg/μl, 2 × 2 μl injections) or equal volume of PBS as described previously^28–30,68^. Rats were anesthetized with isoflurane and received intrastriatal injections (AP + 1.0, ML + 2.0, DV–4.0; AP + 0.1, ML + 4.2, DV–5.0). AP and ML coordinates were measured from bregma, and DV coordinates were measured from dura. Using these intrastriatal PFF coordinates, more than 90% of the resulting α-syn inclusions are localized to the SNpc, with very few inclusions observed in the ventral tegmental area^113^. Injections were performed with pulled glass needles attached to a 10 μl Hamilton syringe^114^, with a flow rate of 0.5 μl min^–1^. To address possible backflow, following each injection, the needle remained in place for 1 min., retracted 0.5 mm, and left in place for an additional 2 min. before removal. Post-surgery, rats received 1.2 mg kg^–1^ of sustained release buprenorphine and were monitored until euthanized on day 60 (2-month) time point.

### Tissue collection

Rats were euthanized with a pentobarbital overdose (Beuthanasia-D Special, Merck Animal Health; 30 mg/kg) until a pedal reflex was absent. Rats were intracardially perfused with ~120 ml of cold heparinized 0.9% saline. Rats designated for pSyn assessment in the SN were additionally perfused with ~150 ml of cold 4% paraformaldehyde in 0.1 M phosphate buffer (pH 7.3). Rats designated for RNASeq or ddPCR endpoints had their brains removed and transferred to a pre-chilled brain matrix on ice, then divided into sections. Sections containing the ipsilateral striatum, ipsilateral SNpc, contralateral striatum, or contralateral SNpc, were flash frozen in 2-methylbutane on dry ice, then stored at –80 °C for later processing. The most rostral sections containing the agranular insular cortex were drop-fixed in 4% paraformaldehyde in 0.1 M phosphate buffer (pH 7.3) for 48 h at 4 °C, then transferred to 30% sucrose for cryoprotection. Rats designated for pSyn assessment in the SN had their whole brain removed and post-fixed in 4% paraformaldehyde in 0.1 M phosphate buffer (pH 7.3) for 48 h at 4 °C, then transferred to 30% sucrose for cryoprotection.

### Immunohistochemistry

Whole brains (or rostral portions in the case of the RNASeq cohort) were mounted on a pre-chilled sliding microtome stage, sectioned to a thickness of 40 µm, and transferred to cryoprotectant. Free-floating sections (1:6 series) were transferred to and washed three times in 0.1 M tris-buffered saline containing 0.5% Triton-X100 (TBS-Tx). Tissue was quenched in 3% H_2_O_2_ for 1 hour, followed by washes with TBS-Tx. Sections were blocked with a 10% normal goat serum in TBS-Tx for 1 hour and incubated overnight at 4 °C in primary antibody (1:10,000 mouse anti-phosphorylated α-syn at serine 129; Abcam, AB184674) in 1% normal goat serum in TBS-Tx. Following washes with TBS-Tx, sections were incubated for 2.5 h at RT in 1:500 goat anti-mouse (Millipore, AP124B) secondary antibody. Sections were washed with TBS-Tx, incubated for 2 h using a standard avidin-biotin complex detection kit (Vector Laboratories, PK-6100), then developed using 2.5 mg ml^–1^ nickel ammonium sulfate hexahydrate (Fisher, N48-500), 0.5 mg ml^–1^ diaminobenzidine (Sigma-Aldrich, D5637) and 0.03% H_2_O_2_ in TBS-Tx. Slides were dehydrated in a graded ethanol series, followed by two exchanges of 100% xylene before cover-slipping with Cytoseal (Thermo-Fisher, 22-050-262). All representative images were acquired with a Nikon Eclipse 90i microscope with a QICAM camera (QImaging, Surrey, British Columbia, Canada), using Nikon Elements AR software (Version 4.500.00, Melville, NY).

### Quantification of pSyn inclusion-bearing neurons in the SNpc

Total enumeration of neurons containing pSyn was performed using Microbrightfield Stereoinvestigator (MBF Bioscience). Sections containing the SNpc (1:6 series) were used for all counts with an investigator blinded to treatment groups. Contours were drawn around the SNpc using the 4X objective. A 20X objective was used to identify stained cells and a marker placed. Stained cells for total enumeration were defined as dark staining either throughout the cell soma or as distinct puncta. The sections used to quantify pSyn were counterstained with cresyl violet to identify neurons and pSyn quantified within cells, so as to not over-estimate cells containing inclusions in the cases where multiple puncta in close proximity are present. Non-specific staining within blood vessels was excluded from the final counts. Total counts for each animal were multiplied by six to estimate the total number of neurons in each animal with inclusions.

### Estrous cycle determination in female rats

Concurrent with the perfusion of the females, vaginal smears were taken to determine the phase of the estrous cycle of each rat. A volume of 50 µl of 0.9% saline was drawn up into a 200 µl pipette tip and the tip inserted into the vaginal canal. The saline was gently dispensed and drawn up in the tip three times, then transferred to a gelatin coated slide. The pipette tip was used to smear the collected fluid across the slide, and the slide allowed to dry completely at RT. Slides were transferred to a Coplin jar containing 0.1% crystal violet for 1 min., washed twice in ddH_2_O, coverslipped, and examined to determine the phases of the estrous cycle^119^. The PBS control group was comprised of three rats in proestrus, one in estrus, and one in metestrus. The PFF group was composed of three rats in proestrus, two in estrus, and one in metestrus (Supplementary Table 3). Rats removed from the study due to low RIN or poor EGFP expression were all coincidentally in proestrus.

### Validation of the α-syn preformed Fibril Model in hTH-GFP rats

Cortical pSyn accumulation, especially in the agranular insular cortex, has previously been observed in rats with efficient nigral pSyn accumulation following intrastriatal PFF injection^25,27–29^. As such, rostral portions containing the agranular insular cortex were stained for pSyn and qualitatively assessed for the presence of inclusions. All PFF injected rats exhibited robust cortical pSyn pathology and could not be excluded from further sample processing (Supplementary Fig. 2).

### Laser capture microdissection

Dissected brain portions containing the ipsilateral SNpc were mounted on pre-chilled cryostat chucks with Tissue-Tek O.C.T. Compound (Sakura, 4583), and frozen on dry ice. Brains were transferred to the cryostat with the chamber temperature set at -19 °C. Brains were allowed to equilibrate for 5 min., then trimmed to remove the majority of the brain surrounding the SN. Using the roll plate, trimmed brains were sectioned to a thickness of 20 µm, then arranged into rows on the chilled stage. Sections through the entire SN were collected on a nuclease and human nucleic acid free polyethylene terephthalate polyester (PET) membrane frame slide (Leica 11505190). Slides were transferred into 50 ml conical tubes containing silica desiccant packets, then buried in dry ice. Sections were immediately used for LCM or stored at –80°C. Frame slides were locked onto the stage of a Leica 6500 Laser Microdissection System. EGFP expressed under the human TH promoter was used to identify and trace a cutting area around the SNpc of each section at 10x magnification. A UV laser (Power = 50, aperture = 7, speed = 7) was used to cut around all the SNpc with set cutting areas on the slide, dropping the tissue into a sample tube cap containing TRIzol reagent (Invitrogen 26696026). One female rat was removed from the study due to poor EGFP expression (Supplementary Table 3). After the programmed cutting cycle, the slides were checked to ensure all cut tissue had dropped. If the tissue did not drop into the TRIzol reagent, the laser was manually used to cut any remaining connections to finish the dissection of the SNpc. In order to preserve RNA integrity, all capture from a slide was performed within 30 min. from the time the slide was removed from dry ice. After the completion of each slide, the collection tube was carefully removed and closed, with the sample remaining in the cap of the tube. In total, we collected 3-4 microdissected sample tubes per brain. Sample tubes were placed on dry ice to freeze the sample, then stored at –80 °C until RNA isolation could be performed.

### RNA Isolation and Sequencing

Phasemaker tubes (Invitrogen, A33248) were labeled and centrifuged for 30 s at 16,000 x *g*. Samples in TRIzol were thawed on ice and briefly centrifuged to collect all the sample at the bottom of the tube. LCM dissected samples from the same brain were all transferred to a single tube, and the total volume brought to 1 ml with TRIzol. Each sample was transferred to a Phasemaker tube, then incubated for 5 min. at RT. For each sample, 200 µL of 100% chloroform was added, the Phasemaker tubes shaken vigorously by hand, incubated for 10 min at RT, then centrifuged for 5 min. at 16,000 x *g* at 4 °C. After centrifugation, the aqueous phase (clear liquid) was transferred to an RNase-free tube, an equal volume of 100% ethanol added, and samples vortexed for 3 s. A column-based nucleic acid purification kit (Zymo Research, R1016) was used with methods modified from the manufacturer’s instructions. The sample was added 600 µL at a time to the column and centrifuged for 1 min at 12,000 x g, until the entire sample had been loaded onto the column. All RNA wash/prep buffer steps were performed by adding the buffer to the column and centrifugation for 1 min at 12,000x*g*. Samples were washed with 400 µl or RNA wash buffer, then a 1X DNase I cocktail (DNase I from Thermo Scientific FEREN0521; Reaction buffer with MgCl_2_ from Thermo Scientific FERB43) was added to the column, incubated for 15 min. at RT. The DNase 1 cocktail was centrifuged through the column, followed by 400 µl of RNA prep buffer, 700 µl of RNA wash buffer, and a final 400 µl of RNA wash buffer. Columns were dried for 2 min. at 12,000 x *g*. DNase/RNase-free molecular grade water (15 µL) was added, columns incubated for 1 min. at RT, and RNA was eluted by centrifugation (1 min. at 10,000 x *g*). Elution was repeated with 10 µl of the flow-through to increase RNA yield. RNA quality and quantity were assessed with an Agilent 2100 Bioanalyzer using an Agilent RNA 6000 Pico Kit (5067-1513), then stored at –80 °C. Prior to RNASeq, 3 males (2 control and 1 PFF) and 2 females (1 control and 1 PFF) were removed due to low RNA integrity number (RIN) post-sample processing (Supplementary Table 3).

### RNA-sequencing library preparation, quality control and quantification

Library preparation and RNASeq were performed at the Van Andel Institute Genomics Core. Libraries were prepared from 10 ng of total RNA using a TruSeq RNA Library Preparation kit ribosomal reduction from Illumina. Paired-end, 2×50 bp sequencing was performed on an Illumina NovaSeq 6000. All libraries were run on a single S2 flowcell with a minimum read depth of 50 M read pairs per library. For all sequencing data, the *FastQC* tool (version 0.11.7) was run on raw.fastq files to generate quality control (QC) plots. During QC, one male control sample was identified as an outlier, and was removed from all downstream analyses. Following QC, read counts were quantified and quasi-mapped to a R*attus norvegicus* index transcriptome using the *salmon* tool (version 0.11.3)^120^. The index transcriptome was generated using the “RTR.fa.gz” file, a comprehensive rat transcriptome available from a recent publication^121^. During read quantification in *salmon*, the *--numBootstraps* parameter was set to 30 bootstraps for each sample. Quantified RNASeq reads were then processed for downstream analyses using the prepare_fish_for_sleuth function in the *wasabi* R package (version 1.0.1)^120^. Linux command line tools and the open-source statistical software R (version 4.0.3) were used for all data processing.

### Differential Transcript Expression Analysis

The *sleuth* R package (version 0.30.0) was used to test wasabi-processed RNASeq data for differential transcript expression (DTE) in PFF-treated animals compared to control^122^. Due to the relatively small sample size available in this study, all DTE models were stratified by sex. Before analysis, the Ensembl rat annotation database was loaded in R using the *biomaRt* package. To avoid inclusion of low-quality data, only transcripts with greater than 10 estimated counts in at least 50% of the samples were included in downstream DTE analyses. After filtering by this count cutoff, DTE models were generated in the *sleuth* package using a combination of the sleuth_prep, sleuth_fit, sleuth_lrt, and sleuth_wt functions. The sleuth_prep function initialized the sleuth object and read in the target mapping and bootstrap information from the provided annotation database and wasabi-processed data, respectively. The sleuth_fit function was then used to produce two smoothed linear models – “full” and “reduced.” In *sleuth*, the “full” model was fit using a smoothed linear model for experimental treatment (control vs. PFF), whereas the “reduced” model was fit assuming equal abundances by treatment. Next, the sleuth_lrt function was used to run a likelihood ratio test (LRT), which identified transcripts with a significantly improved fit in the “full” model compared to “reduced.” Lastly, the sleuth_wt function was used to run a separate Wald test on the abundance data, generating beta values – approximations of log fold change – for each tested transcript. P-values for differential testing were only used from the likelihood ratio test, not the Wald test, as the latter method is less robust for smaller sample size studies. Multiple testing correction was performed using the Benjamini-Hochberg false discovery rate (FDR) method. Given that this was a discovery study, the DTE significance threshold for the *sleuth* LRT results was set to a more lenient FDR-adjusted p-value (or q-value) < 0.2.

### Assessing cell type specificity with DropViz database

To filter down our RNASeq results to only those genes that showed expression in cell types of interest, we accessed DropViz (dropviz.org), a publicly available database that provides transcription data for distinct cell populations in the adult mouse brain^42^. Gene expression and annotation R Data files were downloaded from the DropViz website and loaded into R. Given that DropViz only provides mouse expression data, we used the *biomaRt* package in R to convert our list of differentially expressed rat gene names to mouse gene names. Next, we cross-checked the mouse gene names with the DropViz database, filtering down the list to only those genes that showed expression (Transcripts Per 100,000 > 0) in *Cyp26b1/Anxa1/Grin2c/Vcan*+ neurons, *Myoc/Cst3/Igfbp2*+ astrocytes, *Tmem119*+ microglia, or combined *Myoc/Cst3/Igfbp2/Tmem119*+ astrocytes and microglia. For those grouped classifications that had multiple inclusion criteria (e.g. *Cyp26b1*, *Anxa1*, *Grin2c*, and *Vcan* + ), we included all genes that showed greater than zero expression in any of the selected cell types. DTE results split by cell type with annotated mouse gene names were used for downstream pathway/network analyses.

### Weighted gene correlation network analysis

Weighted gene correlation network analysis (WGCNA) was performed using the *WGCNA* R package (version 1.70-3). The filtered sleuth transcripts per million (TPM) matrix used for differential testing was also used as input for WGCNA. Male and female WGCNA analyses were performed separately to reflect stratified modeling used in differential transcript expression testing. The trait matrix was defined at the experimental group level (e.g. PFF treatment and control). To avoid inclusion of low-quality data, only those transcripts that showed TPM > 1 in all samples were included in WGNCA analysis. The soft-thresholding power was determined by performing a parameter sweep from 1 to 20 and visualizing as a function of scale-free topology fit and mean connectivity. Modules were computed using a soft-thresholding power of 9 for males and 10 for females. For both male and female WGNCA analyses, modules were computed using an unsigned topological overlap matrix (TOM) type, minimum number of module genes set to 30, and a tree cut height of 0.25 to merge modules together. Pearson correlations between module eigengenes and experimental groups were computed considering only pairwise complete observations. Hub genes were determined for each module using a top 10% module membership score cutoff, as has been used in previous literature^44^. Overlap between identified hub genes and differentially expressed transcripts was tested for in both the male and female analyses using the *merge* function in base R, with transcript ID as the merging parameter.

### Pathway analysis

Gene ontology (GO) term enrichment testing and pathway analysis was performed on differentially expressed transcripts using the *clusterProfiler* R package (version 3.18.1)^123^. Pathway analysis was performed on three different sets of results. First, we performed pathway analysis on transcripts that showed differential expression and consistent direction (up- or downregulation) of estimated log fold change by PFF treatment. Second, we performed pathway analysis on transcripts that showed differential expression, consistent direction (up- or downregulation) of estimated log fold change by PFF treatment and overlap with DropViz gene expression results in tissues of interest. For this follow-up, analysis was performed on separate lists of differentially expressed genes that showed expression in specific cell types (neurons, microglia, astrocytes, and microglia+astrocytes). Third, we performed pathway analysis on differentially expressed transcripts that were identified as hub genes using the *WGCNA* package and showed consistent direction (up- or down-regulated) of estimated log fold change by PFF treatment in male and female rats. Within the *clusterProfiler* R package, up- and down-regulated genes were input as separate lists, and the *enrichGO* function was used to perform a hypergeometric over-representation test for gene ontology (GO) term enrichment testing. In a secondary analysis, we also used the *enrichKEGG* function to perform a hypergeometric over-representation test for KEGG term enrichment testing. In all pathway analyses, the Benjamini-Hochberg false discovery rate method was used to correct for multiple testing. Within *clusterProfiler*, the *simplify* function (cutoff = 0.5, by = “p.adjust”) was used to combine redundant terms from GO term enrichment results. For pathway analysis in *clusterProfiler*, significance for GOBP terms was set to FDR-adjusted p-value (or q-value) < 0.05. The *dotplot* and *cnetplot* functions within *clusterProfiler* were used to visualize GO term enrichment analysis results.

### Droplet Digital PCR (ddPCR)

Microdissection of the SN from frozen brains was performed on a cryostat. RNA was isolated from tissue punches using the same methods for the LCM-dissected tissue. Samples were prepared for ddPCR using previously published methods^124^. RNA was thawed, and 2–3 ng was used with iScript Reverse Transcription Supermix for cDNA synthesis (Bio-Rad, 1708841). RNA input was identical for each sample run for an individual transcript of interest, and only varied between transcripts to capture transcripts expressed at lower levels. cDNA synthesis was performed in a thermocycler with the following settings: 5 min. at 25 °C, 20 min. at 46 °C, 1 min. at 95 °C, hold at 4 °C (constant lid temperature of 105°C). In case storage at −20°C was necessary, all cDNA was diluted with 2X cDNA storage buffer (equal parts 10 mM Tris HCl (pH 7.5) and 0.1 mM EDTA pH (8.0)). To prepare samples for droplet digital PCR (ddPCR), cDNA was thawed if needed on ice and master mix containing 2X ddPCR Supermix for Probes (Bio-Rad, 186-3026) and the 20X Taqman primer probe sets were made. Probes used for rats were *Aldh1a1* (Applied Biosystems # Rn00755484_m1, Lot# P230119-000 E06, FAM-MGB), *Bsn* (Applied Biosystems # Rn00569626_m1, Lot# 2041626, FAM-MGB), *Cplx1* (Applied Biosystems # Rn02396766_m1, Lot# 1545259, FAM-MGB), *Cplx2* (Applied Biosystems # Rn01441919_g1, Lot# 1823042, FAM-MGB), *Ddc* (Applied Biosystems # Rn01401189_m1, Lot# 2021653, FAM-MGB), *Drd2* (Applied Biosystems # Rn00561126_m1, Lot# 1970620, FAM-MGB), *Erc2* (Applied Biosystems # Rn00596745_m1, Lot# P230401-000 B05, FAM-MGB), *Nsf* (Applied Biosystems # Rn00572694_m1, Lot# P230401-000 B06, FAM-MGB), *Pclo* (Applied Biosystems # Rn00571796_m1, Lot# Rn00571796, FAM-MGB), *Rab3a* (Applied Biosystems # Rn00564615_m1, Lot# P230401-000 B01, FAM-MGB), *Rab3c* (Applied Biosystems # Rn01493822_m1, Lot# P230401-000 B02, FAM-MGB), *Rab27b* (Applied Biosystems # Rn00584945_m1, Lot# P230401-000 B07, FAM-MGB), *Rgs8* (Applied Biosystems # Rn00571066_m1, Lot# 1616092, FAM-MGB), *Slc6a3* (Applied Biosystems # Rn00562224_m1, Lot# 1771212, FAM-MGB), *Slc18a2* (Applied Biosystems # Rn00564688_m1, Lot# 1728686, FAM-MGB), *Snap25* (Applied Biosystems # Rn00578534_m1, Lot# 1830682, FAM-MGB), *Snca* (Applied Biosystems # Rn00569821_m1, Lot# 1765929, FAM-MGB), *Sncg* (Applied Biosystems # Rn00581652_m1, Lot# 1764532, FAM-MGB), *Stxbp1* (Applied Biosystems # Rn00564767_m1, Lot# P230401-000 B03, FAM-MGB), *Stx1b* (Applied Biosystems # Rn01510167_m1, Lot# P230401-000 B04, FAM-MGB), *Syn1* (Applied Biosystems # Rn00569468_m1, Lot# 1695933, FAM-MGB), *Syn2* (Applied Biosystems # Rn00569739_m1, Lot# 1416478, FAM-MGB), *Syn3* (Applied Biosystems # Rn00567380_m1, Lot# 1581173, FAM-MGB), *Syt1* (Applied Biosystems # Rn00436862_m1, Lot# 1564760, FAM-MGB), *Syt2* (Applied Biosystems # Rn00561994_m1, Lot# 1821869, FAM-MGB), *Syt3* (Applied Biosystems # Rn00569396_m1, Lot# 1821502, FAM-MGB), *Th* (Applied Biosystems # Rn00562500_m1, Lot# 2013779, FAM-MGB), *Vamp2* (Applied Biosystems # Rn01465442_m1, Lot# 1403518, FAM-MGB) and the reference probe was *Rpl13a* (Applied Biosystems # Rn00821946_g1, Lot# P230401-004 H11, VIC-MGB). When available, Taqman primer probe sets used probes which spanned across an exon-exon junction. Equal parts cDNA and master mix were added to tubes, mixed, briefly centrifuged, and 20 µL added to the sample wells of DG8 droplet generator cartridges (Bio-Rad, 1864008). To the oil wells in the cartridge, 70 µL of droplet generation oil (Bio-Rad, 1863005) was added. A rubber gasket (Bio-Rad, 1863009) was secured over the cartridge, and a QX droplet generator (Bio-Rad, 186-4002) used to produce RNA containing droplets. From the droplet well of the cartridge, 40 µL of droplets are transferred to a 96-well plate (Bio-Rad, 12001925). After all samples were transferred, the plate was sealed with pierceable foil (Bio-Rad, 181-4040) by a plate sealer (Bio-Rad, 181-4000). Plates were transferred to a thermocycler (Bio-Rad, C1000), with the following settings: 10 min. at 95 °C, 39 cycles (30 s at 94 °C, 1 min. at 60 °C), 10 min. at 98 °C, hold at 12 °C (constant lid temperature of 105 °C). After PCR, plates were transferred to the QX200 droplet reader (Bio-Rad, 1864003), and results analyzed with QuantaSoft software. For all samples, the gene of interest was normalized to the reference gene. *Rpl13a* was chosen as the reference gene due to its stability and moderate expression in the SN in previous studies^124^. Additionally, the RNA sequencing results show no change in *Rpl13a* expression in the model in either sex (Supplementary File 1). Statistical analysis was performed using GraphPad Prism Version 9.5.0, and significance was performed using α ≤ 0.05. Groups within each sex were analyzed using a two-tailed t-test Outliers were removed using the absolute deviation from the median method^125^, with a difference of 2.5X median absolute deviation used as the exclusion criteria. All group means, standard errors, and statistical test information can be found in Supplementary Data 8.

### Fluorescent in situ hybridization (FISH)

Forty-micrometer-thick coronal brain tissue sections were washed in TBS-Tx and quenched in hydrogen peroxide from the RNAscope Pretreatment Kit (Advanced Cell Diagnostics, Hayward, CA; 322335) for 1 hour. Sections were washed in TBS, mounted on VistaVision HistoBond slides (VWR, Randor, PA; 16004-406), and then placed on a slide warmer at 60°C overnight. Slides were then incubated for 10 min in ACD Biosciences target retrieval buffer (1:10 dilution; Advanced Cell Diagnostics, 322001) at 99 °C and washed twice in water. Tissue was outlined with Pap Pen (Abcam, Cambridge, UK; ab2601), incubated with ACD protease III (Advanced Cell Diagnostics; 322337) in a hybridization oven at 40 °C for 30 mins, washed twice in water, and incubated with the probe for rat *Syt1* (Cat No: 466181, Advanced Cell Diagnostics, Hayward, CA) or for *Slc6a3* (Cat No: 319621, Advanced Cell Diagnostics, Hayward, CA) 2 hours in the hybridization oven at 40°C, followed by washes in ACD wash buffer (1:500 Dilution; Advanced Cell Diagnostics, 310091). Three amplification steps with the amplification buffers (RNAscope™HiPlex12 Detection Reagents (488,550,650) v2; Advanced Cell Diagnostics, 324410) were then performed in 30 min. incubation intervals in the hybridization oven per manufacturer’s instructions. After amplification, slides were washed with RNAscope™Wash Buffer and incubated for 15 min at 40 °C with ACD fluorophores (RNAscope™ HiPlex12 Detection Reagents 550 v2; Advanced Cell Diagnostics, 324410). Slides were washed in RNAscope™Wash Buffer and blocked in 10% normal goat serum in TBX-Tx for 1 hour at room temperature. Sections were incubated with primary antibody (1:10,000 mouse anti-phosphorylated α-syn at serine 129; Abcam, AB184674) in 1% normal goat seurm/TBS-Tx overnight at room temperature. Slides were washed in TBS-Tx and incubated in secondary antibody (1:250 Alexa Fluor 488 goat anti mouse; Invitrogen A32723) for 2 hours at room temperature. Slides were quickly washed 2x in TBS-Tx and slides coverslipped with ProLong™ Gold antifade reagent (Invitrogen, P36930). All samples were imaged with a Nikon Eclipse 90i microscope with a QICAM camera (QImaging, Surrey, British Columbia, Canada).

### Reporting summary

Further information on research design is available in the Nature Research Reporting Summary linked to this article.

### Supplementary information

Supplementary figures and tables

Reporting Summary

Supplementary Data 1

Supplementary Data 2

Supplementary Data 3

Supplementary Data 4

Supplementary Data 5

Supplementary Data 6

Supplementary Data 7

Supplementary Data 8

## Data Availability

All raw RNASeq data have been uploaded in the Gene Expression Omnibus (GEO) data repository (Accession number GSE246112) as per NIH data sharing policy guidelines. Statistical test information for the validation results can be found in Supplementary Data 8. The data that support the findings in this article are available on request from the corresponding author.
