# Supplementary material for: Transcriptomic profiling of early synucleinopathy in rats induced with preformed fibrils
Source: NPJ Parkinsons Dis. 2024 Jan 3;10:7. doi: 10.1038/s41531-023-00620-y (PMC10764951; doi:10.1038/s41531-023-00620-y)
Supplement: Supplementary file 1 — Supplementary figures and tables [file 41531_2023_620_MOESM1_ESM.pdf]

Supplementary Table 1. WGCNA identified transcripts

Transcript ID	Gene/Transcript name	DTE direction	Male			Female		
			q value	b value	standard error (b)	q value	b value	standard error (b)
ENSRNOT00000000814	Ptprc	Up	0.027	1.259	0.174	0.048	0.794	0.124
ENSRNOT00000015198	Laptm5	Up	0.068	0.468	0.109	0.167	0.441	0.124
ENSRNOT00000017385	C1qa	Up	0.146	0.755	0.229	0.064	0.891	0.158
ENSRNOT00000018782	Zc3hav1	Up	0.049	0.893	0.183	0.071	0.516	0.096
ENSRNOT00000025019	Cx3cr1	Up	0.084	0.456	0.113	0.075	0.474	0.094
ENSRNOT00000027360	Mvp	Up	0.055	0.382	0.082	0.074	0.381	0.092
ENSRNOT00000034401	Gfap	Up	0.034	0.874	0.145	0.073	0.841	0.161
ENSRNOT00000037699	Man2b1	Up	0.130	0.286	0.083	0.118	0.293	0.076
ENSRNOT00000051338	Inpp5d	Up	0.105	0.509	0.137	0.064	0.428	0.084
ENSRNOT00000072069	Ctsz	Up	0.079	0.943	0.229	0.062	0.902	0.158
RTRG.2345.1	***Atf5***	Up	0.195	0.292	0.098	0.063	0.640	0.113
RTRG.4893.3	***Unc93b1***	Up	0.120	0.650	0.183	0.049	0.673	0.113
ENSRNOT00000061047	Pcp4	Down	0.021	-0.383	0.042	0.088	-0.380	0.080
ENSRNOT00000079987	Slc6a3	Down	0.048	-0.480	0.097	0.177	-0.336	0.097
ENSRNOT00000083366	Rgs8	Down	0.027	-0.323	0.046	0.098	-0.248	0.066
RTRG.19697.1	***Cplx2***	Down	0.035	-0.378	0.063	0.098	-0.296	0.071
RTRG.37644.2	***Fyb2***	Down	0.068	-0.176	0.049	0.084	-0.259	0.068
ENSRNOT00000011837	Hcn2	Both	0.095	-0.226	0.059	0.075	0.305	0.066
ENSRNOT00000024576	Abhd17a	Both	0.103	-0.234	0.068	0.123	0.277	0.079
ENSRNOT00000044565	Dusp8	Both	0.080	-0.222	0.061	0.109	0.277	0.075
ENSRNOT00000057368	Cdk5r2	Both	0.100	-0.272	0.072	0.072	0.343	0.074
RTRG.32999.11	***Agap3***	Both	0.099	-0.269	0.071	0.195	0.220	0.075

* Genes/transcripts manually annotated are in bold and italicized.

Supplementary Table 2. Comparison between RNASeq and ddPCR

Transcript	Males		Females	
	RNASeq	ddPCR	RNASeq	ddPCR
Aldh1a1	Down	Down	Down	Down
Bsn	Down	Down	No Change	Down
Cplx1	Down	Down	No Change	No Change
Cplx2	Down	Down	Down	Down
Ddc	Down	Down	Down	Down
Drd2	Down	Down	Down	Down
Erc2	Down	Down	Down	Down
Nsf	Down	No Change	No Change	Down
Pclo	Down	Down	Down	Down
Rab27b	Down	Down	No Change	No Change
Rab3a	Down	Down	No Change	No Change
Rab3c	Down	Down	Down	No Change
Rgs8	Down	Down	Down	Down
Rims1	Down	No Change	No Change	Down
Slc18a2	Down	Down	Down	Down
Slc6a3	Down	Down	Down	Down
Snap25	Down	No Change	Down	Down
Snca	Down	Down	Down	Down
Sncg	Down	Down	No Change	Down
Stx1b	Down	Down	No Change	Down
Stxbp1	Down	Down	No Change	Down
Syn1	No Change	Down	No Change	Down
Syn2	Down	Down	No Change	No Change
Syn3	Down	Down	No Change	No Change
Syt1	Down	Down	Down	Down
Syt2	No Change	Down	No Change	No Change
Syt3	No Change	Down	No Change	Down
Th	Down	Down	No Change	Down
Vamp2	Down	No Change	No Change	Down

Supplementary Table 3. Rat treatment, inclusion, and cycle, summary table

Males			Females			
**Treatment**	**Rat**	**Status**	**Treatment**	**Rat**	**Cycle**	**Status**
PFF	1	Included	PFF	12	Metestrus	Included
	2	Included		13	Estrus	Included
	3	Included		14	Estrus	Included
	4	Included		15	Proestrus	Removed (no GFP expression)
	5	Included		16	Proestrus	Included
	6	Removed (low RIN)		17	Proestrus	Removed (low RIN)
PBS	7	Removed (low RIN)	PBS	18	Proestrus	Included
	8	Included		19	Proestrus	Included
	9	Removed (low RIN)		20	Estrus	Included
	10	Included		21	Proestrus	Removed (low RIN)
	11	Included		22	Metestrus	Included

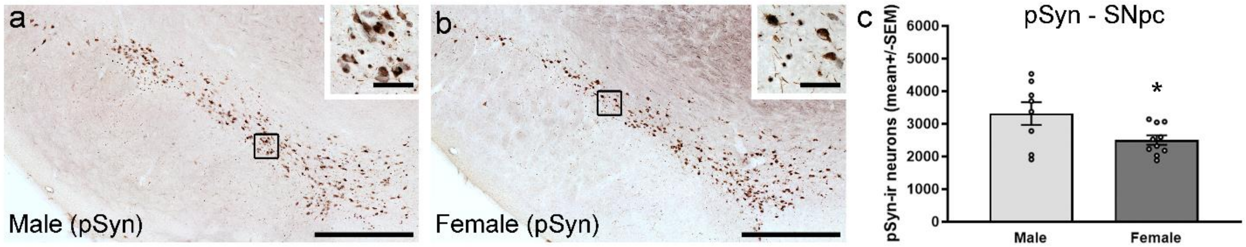

Supplementary Figure 1. Nigral pSyn pathology in male and female rats. Rats received intrastriatal injections of PFFs, and the rostral portion of the brains were fixed and processed for immunohistochemistry 2 months later. Representative images of pSyn in the SNpc in **a.** males and **b.** females. **c.** Total enumeration quantifying pSyn containing neurons shows more pSyn inclusions in males than females. Scale bars are 500 µm in the lower magnification images and 50 µm in the insets. Columns indicate the group means, circles represent individual data points (n=8 per group in males and n=10 per group in females), error bars represent ±1 standard error of the mean. An asterisk represents significance ($p \leq 0.05$).

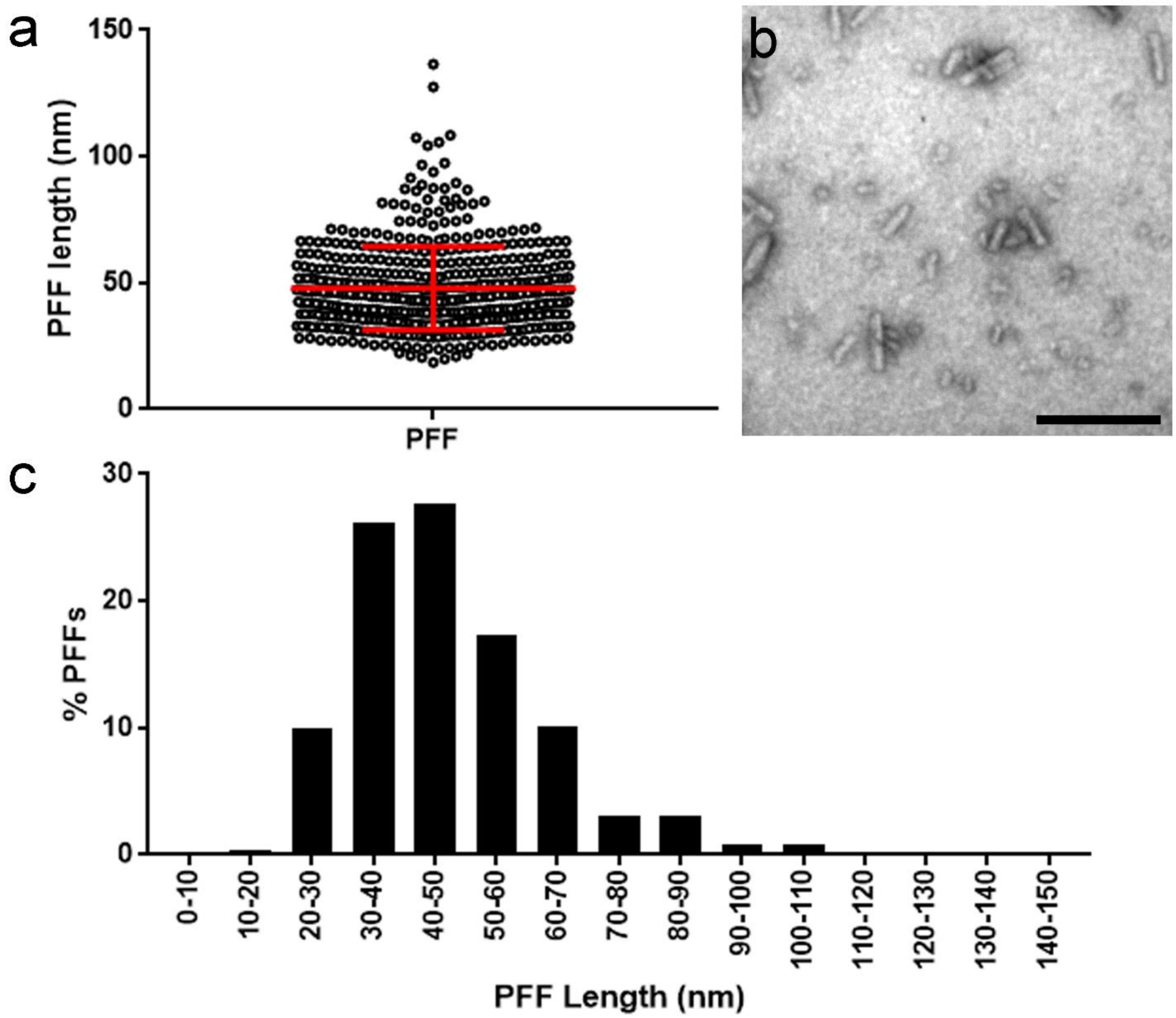

Supplementary Figure 2. Distribution of measured α-synuclein preformed fibril lengths. Post-sonication over 500 preformed fibrils (PFFs) were imaged and measured via transmission electron microscopy. **a.** Lengths of measured PFFs. Each circle represents a measured PFF, the red line denotes the mean, and error bars represent ± 1 standard deviation. **b.** Transmission electron micrograph of sonicated PFFs. Scale bar = 200 nm. **c.** Post-sonication distribution of PFF lengths. Columns represent the percent of PFFs grouped into 10 nm intervals.

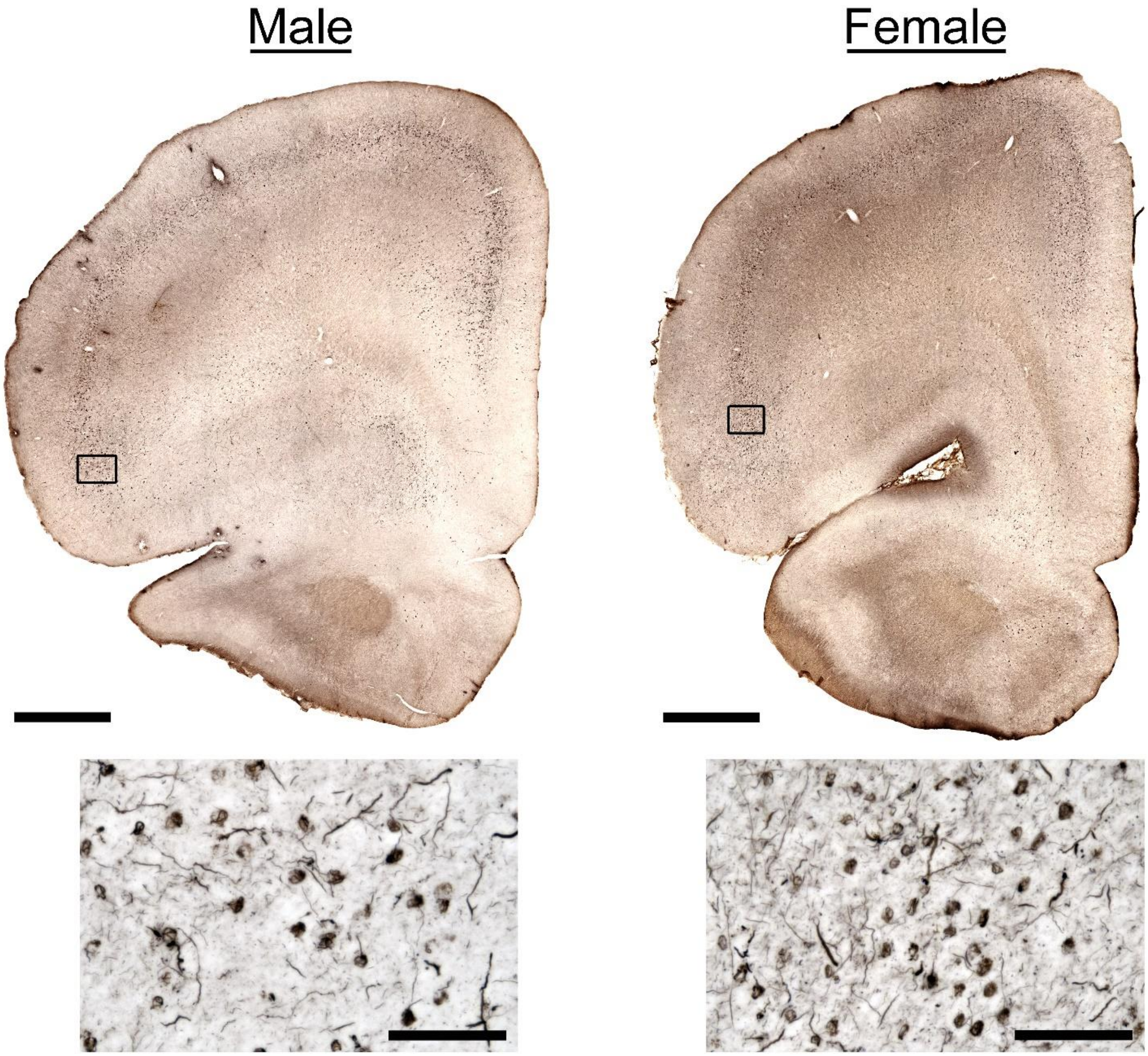

Supplementary Figure 3. Cortical pSyn pathology in male and female rats. Rats received intrastriatal injections of PFFs, and the rostral portion of the brains were fixed and processed for immunohistochemistry 2 months later. Cortical pathology was used to confirm successful seeding in each PFF injected rat. Representative images from the ipsilateral hemisphere from male (**Left**) and female (**Right**) rats are shown. Scale bar = 1 mm. Boxes denote the location of the higher magnification image of the agranular insular cortex, shown below each brain section. Scale bar = 100 µm.
