# Supplementary material for: Transcriptomic profiling of early synucleinopathy in rats induced with preformed fibrils
Source: NPJ Parkinsons Dis. 2024 Jan 3;10:7. doi: 10.1038/s41531-023-00620-y (PMC10764951; doi:10.1038/s41531-023-00620-y)
Supplement: Supplementary file 2 — Reporting Summary [file 41531_2023_620_MOESM2_ESM.pdf]

Corresponding author(s): Joseph R. Patterson
Last updated by author(s): Dec 6, 2023

Reporting Summary

Nature Portfolio wishes to improve the reproducibility of the work that we publish. This form provides structure for consistency and transparency in reporting. For further information on Nature Portfolio policies, see our Editorial Policies and the Editorial Policy Checklist.

Statistics

For all statistical analyses, confirm that the following items are present in the figure legend, table legend, main text, or Methods section.

n/a	Confirmed	
☐	☒	The exact sample size (*n*) for each experimental group/condition, given as a discrete number and unit of measurement
☐	☒	A statement on whether measurements were taken from distinct samples or whether the same sample was measured repeatedly
☐	☒	The statistical test(s) used AND whether they are one- or two-sided<br>*Only common tests should be described solely by name; describe more complex techniques in the Methods section.*
☐	☒	A description of all covariates tested
☐	☒	A description of any assumptions or corrections, such as tests of normality and adjustment for multiple comparisons
☐	☒	A full description of the statistical parameters including central tendency (e.g. means) or other basic estimates (e.g. regression coefficient) AND variation (e.g. standard deviation) or associated estimates of uncertainty (e.g. confidence intervals)
☐	☒	For null hypothesis testing, the test statistic (e.g. *F*, *t*, *r*) with confidence intervals, effect sizes, degrees of freedom and *P* value noted<br>*Give P values as exact values whenever suitable.*
☒	☐	For Bayesian analysis, information on the choice of priors and Markov chain Monte Carlo settings
☒	☐	For hierarchical and complex designs, identification of the appropriate level for tests and full reporting of outcomes
☒	☐	Estimates of effect sizes (e.g. Cohen's *d*, Pearson's *r*), indicating how they were calculated

Our web collection on statistics for biologists contains articles on many of the points above.

Software and code

Policy information about availability of computer code

Data collection	Digital Droplet PCR (ddPCR) data collection was performed with QuantaSoft software version 1.7.4.0917 (BioRad)<br>Images were captured using NIS elements version 4.60, 64 bit (Nikon)
Data analysis	- FastQC tool (version 0.11.7) used to generate quality control plots for raw RNA sequencing data<br>- salmon tool (version 0.11.3) used to align sequences to the rat genome<br>- wasabi R package (version 1.0.1) used to prepare data output for downstream analysis in the sleuth R package<br>- sleuth R package (version 0.30.0) used to identify differential transcript expression<br>- WGCNA R Package (version 1.70-3) used as an unsupervised test to identify hub genes of interest from the RNA sequencing data<br>- clusterProfiler R package (version 3.18.1) used to perform pathway analysis on RNA sequencing data<br>- Graphpad Prism used to perform basic statistical tests

For manuscripts utilizing custom algorithms or software that are central to the research but not yet described in published literature, software must be made available to editors and reviewers. We strongly encourage code deposition in a community repository (e.g. GitHub). See the Nature Portfolio guidelines for submitting code & software for further information.

Data

Policy information about availability of data

All manuscripts must include a data availability statement. This statement should provide the following information, where applicable:

- Accession codes, unique identifiers, or web links for publicly available datasets
- A description of any restrictions on data availability
- For clinical datasets or third party data, please ensure that the statement adheres to our policy

All raw RNASeq data have been uploaded in the Gene Expression Omnibus (GEO) data repository (Accession number GSE246112) as per NIH data sharing policy guidelines. Statistical test information for the validation results can be found in the supplementary statistics document. The data that support the findings in this article are available on reasonable request from the corresponding author.

Research involving human participants, their data, or biological material

Policy information about studies with human participants or human data. See also policy information about sex, gender (identity/presentation), and sexual orientation and race, ethnicity and racism.

Reporting on sex and gender	NA
Reporting on race, ethnicity, or other socially relevant groupings	NA
Population characteristics	NA
Recruitment	NA
Ethics oversight	NA

Note that full information on the approval of the study protocol must also be provided in the manuscript.

Field-specific reporting

Please select the one below that is the best fit for your research. If you are not sure, read the appropriate sections before making your selection.

☒ Life sciences ☐ Behavioural & social sciences ☐ Ecological, evolutionary & environmental sciences

For a reference copy of the document with all sections, see nature.com/documents/nr-reporting-summary-flat.pdf

Life sciences study design

All studies must disclose on these points even when the disclosure is negative.

Sample size	For the RNA sequencing experiment, power analysis was performed by the Van Andel Institute Genomics Core to be able to identify a 50% change. For the confirmatory ddPCR experiments, power calculations were performed in SigmaPlot 12.0, where significance is $\alpha \leq 0.05$ and power is $\beta \geq 0.80$. Sample sizes selected allow for the exclusion of two rats per group (missed injection, death, etc.) while still maintaining a power of $\beta \geq 0.80$. For all experiments listed, the estimated standard deviation is 15%, based on previous ddPCR data from our group.
Data exclusions	For the RNA sequencing experiment, outliers were removed due to lack of GFP expression (could not identify the outline of the substantia nigra when performing laser capture microdissection), sample RNA integrity was too low for sequencing, and low quality RNA reads based on FastQC plots. These omissions of animals are outlined in supplementary table 3. For the confirmatory ddPCR experiments, Outliers were assessed using the absolute deviation from the median method (Leys et al., 2013), with a "very conservative" difference of 2.5X median absolute deviation used as the exclusion criteria.
Replication	A subsets of transcripts of interest from the RNA sequencing results were examined using ddPCR (primer specific) in an additional cohort of non-transgenic animals which were generated using different batches of PFFs and during a different surgical session. How the ddPCR and RNA sequencing data compare for these specific transcripts is shown in supplementary table 2.
Randomization	Animals were randomly assigned treatment groups by cage at the beginning of the experiments.
Blinding	Animals were randomly assigned to treatment groups and assigned a number. Identities of groups were not reveled to those performing the assessments.

Reporting for specific materials, systems and methods

We require information from authors about some types of materials, experimental systems and methods used in many studies. Here, indicate whether each material, system or method listed is relevant to your study. If you are not sure if a list item applies to your research, read the appropriate section before selecting a response.

Materials & experimental systems

n/a	Involved in the study
☐	☒ Antibodies
☒	☐ Eukaryotic cell lines
☒	☐ Palaeontology and archaeology
☐	☒ Animals and other organisms
☒	☐ Clinical data
☒	☐ Dual use research of concern
☒	☐ Plants

Methods

n/a	Involved in the study
☒	☐ ChIP-seq
☒	☐ Flow cytometry
☒	☐ MRI-based neuroimaging

Antibodies

Antibodies used	Mouse anti-α-syn phosphorylated at serine 129 (Abcam, ab184674 Lot: 1013051-1)<br>Goat anti-mouse (Millipore, AP124B Lot:3862021)
Validation	Mouse anti-α-syn phosphorylated at serine 129 (Abcam, ab184674) product and validation information from the company can be found at https://www.abcam.com/products/primary-antibodies/alpha-synuclein-phospho-s129-antibody-p-syn81a-ab184674.html. Additionally, we have published with this antibody for years and observe it is specific for Lewy body-like inclusions induced by alpha-synuclein preformed fibrils in rodents.

Animals and other research organisms

Policy information about studies involving animals; ARRIVE guidelines recommended for reporting animal research, and Sex and Gender in Research

Laboratory animals	For RNASequencing:<br>- Three-month-old, male hTH-EGFP rats (n=11) were purchased from Taconic Biosciences (Taconic #12141; NTac:SD-Tg (TH-EGFP) 24Xen)<br>- Three-month-old, female hTH-EGFP rats (n=11) were purchased from Taconic Biosciences (Taconic #12141; NTac:SD-Tg (TH-EGFP) 24Xen)<br><br>For ddPCR:<br>- Three-month-old, male Fischer 344 rats (n=16 were purchased from Charles River Laboratories.<br>- Three-month-old, female Fischer 344 rats (n=16) were purchased from Charles River Laboratories.<br><br>For comparison of phospho-alpha-synuclein (pSyn S129) between sexes and fluorescent in situ hybridization (FISH):<br>- Three-month-old, male Fischer 344 rats (n=16 were purchased from Charles River Laboratories.<br>- Three-month-old, female Fischer 344 rats (n=20) were purchased from Charles River Laboratories.
Wild animals	NA
Reporting on sex	Sex as a variable was taken into account in the study design of the the RNA sequencing, ddPCR, and comparison of pSyn inclusions in the substantia nigra. Throughout the manuscript we report the differences in each sex in our text, figures, and tables.
Field-collected samples	NA
Ethics oversight	All procedures were approved and conducted in accordance with the Michigan State University Institute for Animal Care and Use Committee (IACUC) at Michigan State University. Procedures under the RNA sequencing study were approved by the Animal Care and Use Review Office (ACURO) of the United States Army Medical Research and Development Command Office of Research Protections.

Note that full information on the approval of the study protocol must also be provided in the manuscript.

Plants

Seed stocks	NA
Novel plant genotypes	NA
Authentication	NA
